# The Regulatory Army of Plant Defense: Transcription Factors in the War for Plant Immunity

**DOI:** 10.3390/ijms27167315

**Published:** 2026-08-16

**Authors:** José Ribamar Costa Ferreira-Neto, Agnes Angélica Guedes de Barros, Ana Luíza Trajano Mangueira de Melo, Lidiane Lindinalva Barbosa Amorim, Madson Allan de Luna Aragão, João Pacífico Bezerra-Neto, Laiane Silva Maciel, Manassés Daniel da Silva, Paulo Vitor Galdino da Silva, Ana Maria Benko-Iseppon

**Affiliations:** 1 Laboratory of Plant Genetics and Biotechnology, Department of Genetics, Biosciences Center, Federal University of Pernambuco, Recife 50.740-600, Pernambuco, Brazil; agnes.barros88@gmail.com (A.A.G.d.B.); laianesm@gmail.com (L.S.M.); manasses.dsilva@ufpe.br (M.D.d.S.);; 2 Federal Institute of Piauí-Campus Pedro II, Pedro II 64.255-000, Piauí, Brazil; 3 Institute of Biological Sciences–ICB, Federal University of Minas Gerais, Belo Horizonte 31.270-901, Minas Gerais, Brazil; 4 University of Pernambuco-Campus Petrolina, Petrolina 56.328-900, Pernambuco, Brazil; pacifico.joao@upe.br

**Keywords:** pattern-triggered immunity, effector-triggered immunity, transcriptional reprogramming, hormone signaling

## Abstract

Plant diseases impose major constraints on global crop productivity and pose a major threat to food security. Here, we review transcription factors (TFs) as central orchestrators of plant defense, consolidating recent advances in how these regulators connect pathogen perception to immune signaling, transcriptional reprogramming, and durable defense responses. Initially, we combined a literature-based synthesis with a natural language processing (NLP) analysis of 1647 PubMed abstracts published between 2021 and 2026 to map dominant and underexplored TF families associated with plant immunity. WRKY, MYB, AP2/ERF, bHLH/MYC, and NAC dominated the recent literature, whereas families such as NF-Y, Trihelix, PLATZ, TCP, and GRAS represent emerging regulatory actors. Across these and other families, TFs integrate pattern- and effector-triggered immunity, hormone crosstalk, chromatin dynamics, non-coding RNA regulation, post-translational modifications, and metabolic remodeling, in addition to cell-type-specific expression. Further evidence indicates that pathogens frequently manipulate TFs to weaken host defense, underscoring their central position in plant molecular physiology and plant-pathogen coevolution. The data emphasize that TF function is context-dependent and influenced by multilayered regulation, cell type, pathogen lifestyle, and host genetic background. This review provides a framework for understanding TFs in plant immune control and highlights TF-centered strategies for engineering durable crop resistance, along with future challenges.

## 1. Introduction

One of the clearest ways in which plant health affects human well-being is through food security. Crop losses from pests and diseases directly undermine agricultural productivity and reduce the food supply. Fungal pathogens, oomycetes, bacteria, viruses, nematodes, and insect herbivores cause losses of billions of dollars per year in tropical and subtropical regions [1]. According to estimates from the Food and Agriculture Organization [2], these losses account for nearly 40% of global crop production annually. This burden falls most heavily on vulnerable populations, particularly those already exposed to food insecurity driven by climate change, economic instability, or armed conflict [2].

In human-managed agroecosystems, pests and pathogens are managed through a dynamic combination of ecological knowledge, technological innovation, and coordinated governance. Historically, humans have relied on sanitation, quarantine, crop rotation, biological control, and integrated pest management to reduce yield losses [3,4]. Pesticides, however, remain a central tool for protecting crops against biological threats; their intensive and recurrent use has generated substantial agronomic, environmental, and public health concerns [5,6].

Beyond the human-centered perspective outlined above, plants themselves play crucial strategic roles in ensuring their survival. In response to the biotic threats mentioned, plants have evolved a multilayered defense system that detects and responds to pathogen attacks with high specificity and speed. This system is described as a two-layered mechanism [7]. The first layer, called pattern-triggered immunity (PTI), is activated when cell-surface pattern recognition receptors (PRRs) perceive conserved pathogen-associated molecular patterns (PAMPs, such as bacterial flagellin and fungal chitin). This perception induces broad defense responses, including mitogen-activated protein kinase (MAPK) signaling and transcriptional reprogramming of defense genes [7]. The second layer, termed effector-triggered immunity (ETI), is mediated by intracellular nucleotide-binding leucine-rich repeat (NLR) receptors that recognize pathogen effectors (i.e., virulence factors delivered into host cells to suppress PTI), either directly or by sensing perturbations in host target proteins [8].

Central to the execution of both PTI and ETI mechanisms is the rapid and large-scale transcriptional reprogramming of the plant genome. Within minutes to hours after pathogen perception, thousands of genes are differentially expressed (up- and down-regulated). This reprogramming triggers PTI and ETI outputs, including signal transduction pathways, cell wall reinforcement machinery, antimicrobial compounds such as phytoalexins, pathogenesis-related (PR) proteins, and regulators of hormone signaling [7,8,9]. The precision and timing of this transcriptional response are essential determinants of resistance outcome, plant survival, and adaptation. These gene-expression parameters are governed by transcription factors (TFs). TFs bind cis-regulatory DNA sequences and recruit or repel the basal transcriptional machinery. This machinery comprises the minimum set of protein factors required for RNA polymerase to initiate transcription at a core promoter. Through these activities, TFs act as master regulators of gene activity.

Research on plant defense-related TF families has expanded rapidly, driven by advances in molecular genetics, multi-omics technologies, bioinformatics, and biotechnology. High-throughput approaches such as ChIP-Seq (chromatin immunoprecipitation followed by sequencing) [10], single-cell transcriptomics [11], transgenic and functional genomics studies [12], genome-wide association studies (GWASs) [13], and CRISPR/Cas9-mediated genome editing [14], among others, have established TFs as central regulators of plant immunity. These studies have demonstrated that TFs not only orchestrate defense activation in a tissue- and cell-type-specific manner but also represent major targets of pathogen effectors that disrupt host transcriptional networks.

Despite the aforementioned advances, a comprehensive synthesis of the regulatory landscape governing defense-related TFs remains lacking. Existing reviews have generally focused on individual TF families (e.g., NAC [15], MYB [16]), addressed transcriptional regulation only as part of broader discussions on plant immunity [17], examined TFs in specific biological contexts such as secondary metabolite biosynthesis [18] or responses to particular pathogen groups (e.g., viruses [19]), or adopted a broader perspective encompassing both abiotic and biotic stresses [20,21], rather than focusing specifically on plant-pathogen interactions. In 2017, our research group published a comprehensive review on the role of TFs in plant defense [22], summarizing the then-current understanding of their structure, regulatory mechanisms, signaling pathways, and protein-protein interactions. That review emphasized only the ERF, bZIP, and WRKY families because of their abundance, biological relevance, and the availability of functionally characterized members associated with disease resistance [22]. The present review moves beyond the family-centered perspective by integrating a decade of conceptual and technological advances into a framework for understanding TF-mediated plant immunity. Unlike previous reviews, we jointly examine major and emerging TF families involved in plant responses to pathogens; current knowledge on the regulatory mechanisms that enable TFs to act with precision, efficiency, and context-dependent specificity; their cell-type-specific behavior as revealed by single-cell and spatial transcriptomics approaches; how these proteins are targeted by pathogenic microorganisms seeking to weaken plant defenses and facilitate infection; and translational biotechnological strategies aimed at converting the theoretical framework surrounding the “TFs and plant defense” interface into practical interventions that enhance plant performance under pathogen pressure. Finally, we discuss a TF-centered framework for plant immunity, future perspectives, outstanding bottlenecks, and key challenges that must be overcome to advance this rapidly evolving field.

By bridging molecular mechanisms; cutting-edge, multifaceted knowledge; agronomic applications; and forward-looking perspectives on the topic addressed herein, this review seeks to serve as a comprehensive reference for researchers working across plant molecular biology, functional genomics, and applied crop science.

## 2. The Known and the Emerging: Core and Underexplored Transcription Factor Families in Plant Defense

The content presented in the following Section 2.1 and Section 2.2 was derived from a natural language processing (NLP)-based analysis to quantify the current research landscape on TFs associated with plant defense. To this end, a corpus of 1647 abstracts was compiled from the PubMed database (https://pubmed.ncbi.nlm.nih.gov/; accessed on 1 April 2026) using the query “transcription factors AND plant defense NOT review”, with the search restricted to articles published within the last five years (2021–2026). The full information-processing pipeline underlying this analysis is provided in Supplementary Material S1, and the analyzed corpus is presented in Supplementary Material S2.

### 2.1. Established Pillars in Plant Defense: The Major Transcription Factor Families

**Figure 1:**
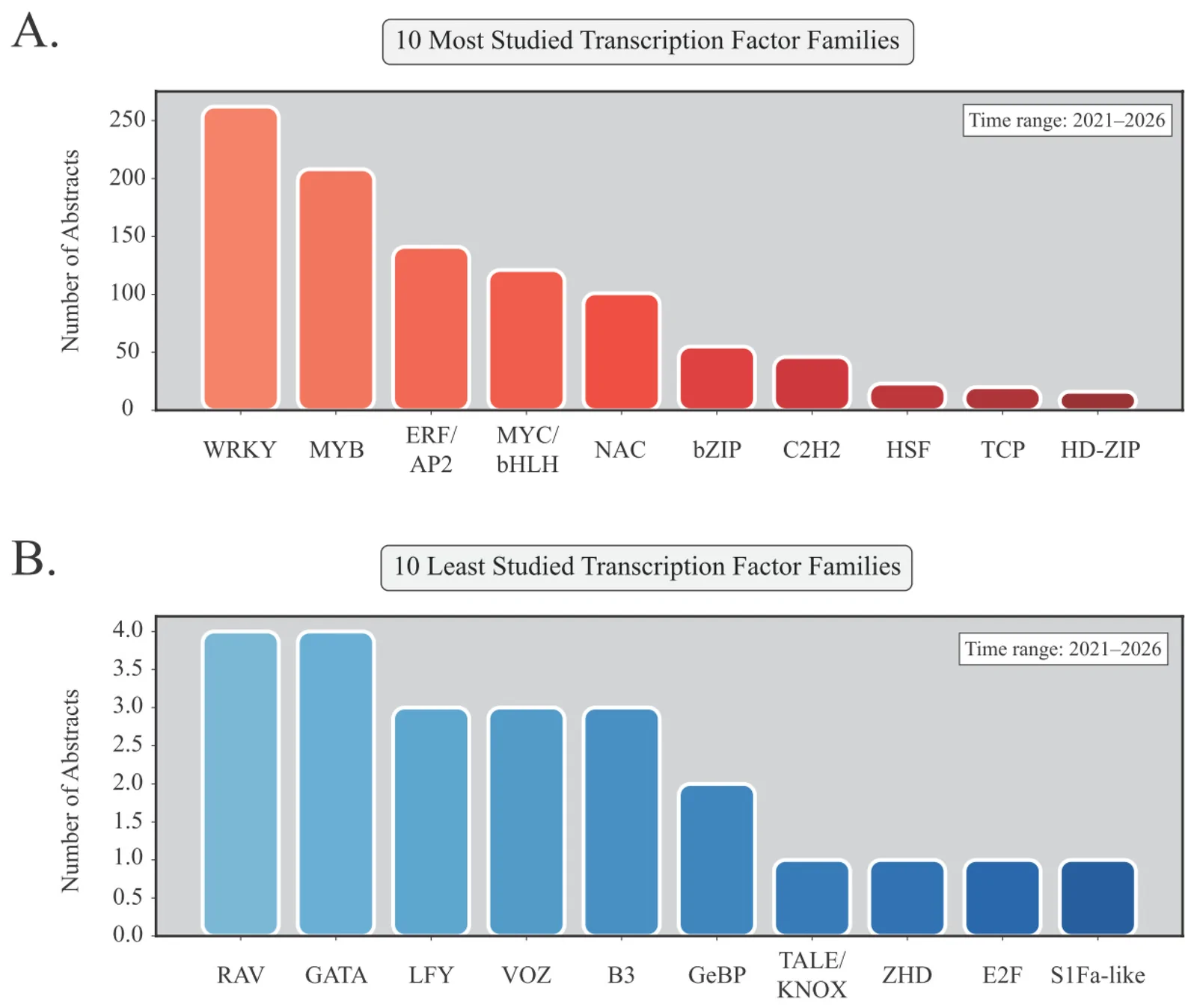
Quantification * of the 10 most (**A**) and 10 least (**B**) studied transcription factor (TF) families in the context of plant defense, based on the analysis of a textual corpus comprising 1647 abstracts from studies published between 2021 and May/2026 and available in the PubMed database. The bar plots show the number of studies addressing each transcription factor family. * The document frequency metric adopted here reflects the breadth of scientific investigation directed at each TF family within the defined corpus, and should not be interpreted as a direct proxy for biological relevance or functional importance. TF families that currently remain underrepresented in the literature may nonetheless emerge as critical regulatory nodes in plant immunity as future research further elucidates their functions.

The NLP analysis identified WRKY, MYB, ERF/AP2, MYC/bHLH, NAC, bZIP, Zinc Finger (C2H2/ZF), HSF, TCP, and HD-ZIP as the ten TF families most extensively investigated in plant defense over the last quinquennium (Figure 1).

From this broader set, we selected five of the best-characterized TF families for in-depth discussion, with the aim of highlighting major research trends and evaluating the extent to which available experimental evidence supports the functional models proposed for each group. The following sections examine recent studies to clarify the mechanisms of action of these TFs, as well as the regulatory networks they establish with other molecular components involved in plant defense.

#### 2.1.1. WRKY Transcription Factors

The WRKY family constitutes the most thoroughly investigated class of defense-related TFs in plants, with 262 studies addressing its roles and functions (Figure 1; Table 1). At the broadest level, recent reports indicate that some WRKYs function as transcriptional activators and repressors of defense-related genes, and their collective action encompasses PTI and ETI layers.

In the context of PTI, some WRKYs emerge as key downstream regulators of PAMP signaling. For instance, rapeseed *BaWRKY53* promotes PTI by directly activating the salicylic acid (SA) biosynthetic genes *ICS1* and *PBS3*, thereby amplifying SA-dependent defense signaling in response to *Pseudomonas syringae* challenge [23]. Conversely, phytosulfokine, a growth-promoting peptide hormone, attenuates PTI by downregulating a suite of defense-associated WRKY genes in *Arabidopsis*, revealing that these TFs occupy a node of convergence between immunity and growth signaling [24]. In ETI, *NbWRKY7* in *Nicotiana benthamiana* was shown to directly bind W-box elements in the *NRG1* promoter, a key helper NLR gene, thereby enhancing both basal immunity and TNL-mediated resistance (ETI). Notably, activated *NRG1* resistosomes, in turn, further induce *NbWRKY7* transcription, establishing a positive feedback loop that amplifies the immune signal [25].

The mechanistic basis for WRKY activity is intimately connected to post-translational regulation by MAPK cascades. Phosphorylation by MAPKs enhances the activity of specific WRKY proteins, enabling rapid and robust induction of defense gene expression. In oilseed rape (*Brassica napus*), the *BnaA03.MKK5-BnaA06.MPK3/BnaC03.MPK3* module phosphorylates *BnWRKY33* in the early stages of *Sclerotinia sclerotiorum* infection, triggering a transcriptional burst of *BnWRKY33* itself that drives phytoalexin biosynthesis [26]. A parallel mechanism operates in cotton (*Gossypium hirsutum*), where the Raf-like kinase *GhRAF39_1* acts as a helper that facilitates *GhMPK9*-mediated phosphorylation of *GhWRKY40a. The activated WRKY TF* then converges on *GhERF1b*- and *GhABF2*-mediated pathways to coordinate transcriptional and stomatal defense against the fungus *Verticillium dahliae* [27].

WRKYs are not only regulated by upstream kinases but are also embedded in complex transcriptional networks characterized by both cooperative and antagonistic interactions among family members. WRKY-WRKY regulatory modules are a recurrent theme: in pepper (*Capsicum annuum*), *CaWRKY28* lacks direct W-box-binding activity. Still, it potentiates *CaWRKY40*-mediated activation of immunity genes, including *CaPR1*, *CaNPR1* (a transcriptional coactivator that interacts with TFs to trigger defense gene expression), and *CaDEF1*, through physical protein-protein interaction in response to the bacterium *Ralstonia solanacearum* [28]. In tomato, *SlWRKY44* and *SlWRKY48* antagonistically regulate *SlWRKY30IIc* expression (a positive regulator of resistance to *Meloidogyne incognita*) by competing for the same promoter-binding site, with *SlWRKY44* promoting and *SlWRKY48* suppressing resistance [29]. Notably, *SlWRKY44* physically interacts with *SlWRKY48*, and the two factors competitively modulate *SlWRKY30IIc* expression [29].

WRKY TFs also function as central hubs integrating immune signaling with phytohormone pathways, particularly salicylic acid (SA)- and jasmonate (JA)-mediated defenses. For example, the SA–WRKY70–PR–callose regulatory module was recently shown to mediate tobacco (*Nicotiana tabacum*) resistance against whitefly (*Bemisia tabaci*) oviposition: *NtWRKY70a* activates *NtPR1-L1* expression, which in turn promotes callose deposition that physically impedes egg access to water and nutrients [30]. In the JA pathway, *GbWRKY11* in cotton (*Gossypium barbadense*) directly binds to the W-box in the *GbLOX5* (a key JA biosynthesis gene) promoter, activating JA biosynthesis and thereby conferring resistance to *V. dahliae* [31]. These hormone-linked functions extend across diverse plant-pathogen systems and multiple species.

Evidence from other functional studies reinforces the idea that WRKY-mediated immunity is mechanistically diverse. In *Ocimum sanctum*, *OscWRKY1* was shown to bind W-box elements in the promoters of phenylpropanoid pathway genes such as *PAL* and *C4H*, positively regulating their expression and enhancing resistance to *P. syringae* pv. *tomato* DC3000 in transgenic *Arabidopsis* [32]. This study is particularly informative because it links WRKY activity not only to canonical immune signaling but also to metabolic reinforcement of defense through the phenylpropanoid pathway. In turn, Wen et al. [33] characterized *AktWRKY12* from *Akebia trifoliata* as a pathogen-induced WRKY whose heterologous overexpression suppressed lignin biosynthetic genes in tobacco plants, suggesting that some WRKY members may act as negative regulators of defense-associated structural barriers. Together, these findings illustrate a key principle in WRKY biology: their contribution to resistance is strongly context-dependent, varying with target genes, host species, and pathosystems.

#### 2.1.2. MYB Transcription Factors

The MYB superfamily was the second most frequently addressed TF group in the analyzed dataset, being discussed in 208 scientific articles (Figure 1; Table 1). The R2R3-MYB class is by far the most functionally characterized and most associated with biotic stress responses (Supplementary Material S2).

In the current literature, a dominant theme linking MYBs to plant immunity is their role in activating lignin biosynthesis. Lignin deposition at sites of pathogen attack constitutes a conserved physical barrier that restricts hyphal penetration and colonization. Multiple independent studies document R2R3-MYB members as direct transcriptional activators of lignin biosynthetic genes. In chrysanthemum (*Chrysanthemum morifolium*), *CmMYB15-like* is induced by aphid (*Macrosiphoniella sanborni*) feeding and directly binds AC cis-elements in the promoter of the gene *Cm4CL2* (encoding a key enzyme of the lignin biosynthesis pathway), promoting lignin deposition and reducing aphid damage [34]. The cotton (*G. hirsutum*) gene *GhODO1* enhances resistance to *V. dahliae* by binding to the promoters of the *Gh4CL1* and *GhCAD3* genes, directly activating their expression and increasing total lignin accumulation. Silencing GhODO1 leads to reduced lignin and increased disease susceptibility [35]. In pear (*Pyrus* spp.), *PcMYB44* activates lignin biosynthesis via regulation of the *PcmiR397*-*PcLACs* module. It directly induces disease-resistance genes in the JA, SA, and ethylene (ET) pathways, conferring protection against *Botryosphaeria dothidea* ring rot [36]. The rice (*Oryza sativa*) MYB factor *OsMYB30*, in turn, operates within a WRKY-MYB hierarchical module. OsWRKY47 transcriptionally activates *OsMYB30* in the early stages of *Magnaporthe oryzae* infection to reinforce cell wall lignification and prevent fungal penetration. A separate WRKY node simultaneously deploys chemical defenses (i.e., accumulation of stevioside, a metabolite that activates the plant immune response) at later infection stages [37]. In *N. benthamiana*, several R2R3-MYB genes are strongly induced by whitefly infestation [38]. Overexpression of *NbMYB42*, *NbMYB107*, *NbMYB163*, *NbMYB247*, and *NbMYB423* induced the expression of key genes in lignin biosynthesis and SA-signaling pathways, contributing to a dual-layered chemical and signaling defense [38].

MYBs are embedded in intricate transcriptional networks that frequently intersect with growth-regulatory circuits. An illustrative case is GhMYB33 in cotton (*G. hirsutum*), identified as a hub gene balancing growth and defense; it interacts with the DELLA protein *GhGAI1*. It binds the promoters of both an internode elongation gene (*GhSPL9*) and an anthocyanin biosynthesis gene (*GhDFR1*), mediating the trade-off between biomass and resistance to the fungus *V. dahliae* [39]. Additionally, the miRNA ghr-miR319c was discovered to target and suppress *GhMYB33* expression [39]. Another growth-defense coordination mechanism has also been reported for the MYB-related TF *SRM1* in *Arabidopsis*, which positively affects both immunity (by directly activating the *EDS1* gene and anthocyanin biosynthesis) and growth (by activating chlorophyll synthesis) [40]. Upon pathogen infection, SRM1 is stabilized via MPK3/6-mediated phosphorylation, preventing its proteasomal degradation and increasing its stability and accumulation [40]. Interactions with other TF families further extend the MYB regulatory network: in grapevine (*Vitis amurensis*), VaMYB306 does not bind defense gene promoters independently but forms a hetero-complex with VaERF16 that synergistically activates *VaPDF1.2* (a defensin-like protein) and enhances resistance to *Botrytis cinerea* [41].

The body of evidence supporting functional models for MYB factors in plant defense is substantial and methodologically diverse. The prevailing model, in which R2R3-MYB factors serve as hormone-responsive transcriptional activators and regulators of cell wall reinforcement pathways, is well supported across multiple plant-pathogen systems in the current scientific literature.

#### 2.1.3. ERF/AP2 Transcription Factors

The AP2/ERF superfamily ranked as the third most extensively investigated group in TF-plant defense associations, constituting the primary subject of analysis in 141 studies (Figure 1; Table 1). The superfamily is subdivided into four main groups: AP2, RAV, DREB, and ERF, with ERFs constituting the largest subgroup [42].

In the current literature on TFs and plant defense, the mechanistic basis of ERF activity in immunity is best understood in the context of the ET/JA defense pathway, which governs resistance against necrotrophic pathogens and herbivores [43]. The Arabidopsis AP2/ERF *ORA59* is a major positive regulator of this pathway. It directly binds GCC-box elements in the promoter of the *AtACT* (*agmatine coumaroyltransferase*) gene to drive the production of hydroxycinnamic acid amides, antimicrobial phytoalexins effective against necrotrophs [44]. Apple (*Malus × domestica*) *MdERF100*, in turn, also provides a relevant example of the AP2/ERF impact on plant physiology. Heterologous overexpression of MdERF100 in *Arabidopsis* enhanced resistance to powdery mildew (caused by *Podosphaera leucotricha*) through modulation of JA and SA signaling. This defense effect was potentiated by physical interaction with the bHLH co-factor *MdbHLH92*, highlighting how ERF activity is frequently executed through multi-protein complexes [45].

Another prominent mechanistic theme is the positioning of some ERF factors as endpoints of MAPK signaling cascades. In cotton, the *GhMPK9-GhRAF39_1-GhWRKY40a* phosphorylation module converges on the ERF *GhERF1b*. The phosphorylated GhWRKY40a activates GhERF1b transcription, which in turn induces defense-related genes, while simultaneously suppressing stomatal aperture through *GhABF2*, effectively coupling transcriptional and physical barriers against *V. dahliae* [27]. ET-signaling components upstream of ERFs also directly regulate immunity [46]. The EIN3-type TF *GmEIL1* in soybean (*Glycine max*) is induced by *Phytophthora sojae*, directly activates *GmERF113* and *GmPR1*, and confers resistance through the ET pathway. Silencing of *GmEIL1* markedly increased disease severity, establishing the EIN3-ERF module as a functional immunity axis [46].

ERFs are also embedded, directly or indirectly, in networks that balance immunity against (hemi)biotrophic and necrotrophic pathogens, often through reciprocal SA-JA regulation. The rice EIN3-like factor *OsEIL2* illustrates this duality with particular clarity. Upon necrotrophic *Rhizoctonia solani* challenge, *OsEIL2* binds the *OsWRKY67* promoter to induce SA/JA pathway genes and enhance resistance. By contrast, upon biotrophic and hemibiotrophic challenge (by *Xanthomonas oryzae* pv. *oryzae* and *M. oryzae*, respectively), *OsEIL2 targets OsERF083* to suppress the same SA/JA pathway, thereby increasing susceptibility. These findings show that the same EIN3-like TF can act as either a positive or negative immune regulator depending on pathogen lifestyle [47]. *ERF3* in *Arabidopsis* plays an analogous antagonistic role. It is pathogen-induced and represses the SA defense pathway by suppressing *PR2*, *PR5*, and defense-associated WRKY genes. Consistent with this role, erf3 loss-of-function mutants showed enhanced resistance to *P. syringae* pv. *tomato* DC3000. Conversely, ERF3 overexpressors were hypersusceptible [48]. The ERF group VII (ERFVII) subclass, in turn, has recently emerged as an unexpected contributor to immunity in an interesting context. Soybean GmERFVII members *GmRAP2.12* and *GmRAP2.3*, canonically known as hypoxia sensors regulated through the N-end rule protein degradation pathway, directly activate the *GmPR10* gene (GmPR10-09g). This PR10 family member is highly induced by soybean cyst nematode (SCN; *Heterodera glycines*) infection and positively contributes to nematode resistance [49]. Hypoxic pretreatment, which stabilizes ERFVIIs, also primes SCN resistance, suggesting that the oxygen-sensing machinery is coopted for biotic defense [49].

These findings position AP2/ERF TFs as versatile regulatory hubs in plant immunity, integrating hormonal, MAPK, oxygen-sensing, and pathogen-specific signals. Their context-dependent activity underscores both the complexity of defense regulation and the potential of ERF-centered networks for engineering durable stress resilience in crops.

#### 2.1.4. bHLH/MYC Transcription Factors in Plant Defense

Within the broad bHLH family, the fourth most studied family (121 studies; Figure 1; Table 1), the MYC subgroup, occupies a uniquely central position in plant immunity. MYC proteins function as transcriptional hubs that convert JA perception into defense-associated transcriptional reprogramming, particularly in wound- and herbivory-related responses, while also contributing to the broader modulation of JA-dependent immunity [50,51]. Rather than acting as simple on/off switches for defense, some MYC TFs coordinate immune metabolism, developmental restraint, and resource allocation, thereby shaping the classical growth-defense trade-off [52,53]. This role is especially evident in *Arabidopsis*, where MYC TFs promote resistance to insect herbivory but simultaneously reduce shoot and root growth, fruit size, and seed yield under constitutively elevated defense states [54]. Similar growth-defense trade-offs are observed in *Nicotiana attenuata*, where silencing of *NaMYC2a/b* by RNAi (RNA interference) increases fitness under reduced herbivore pressure but causes severe susceptibility under high herbivore load because of reduced accumulation of specialized metabolites [55].

Mechanistically, MYC activity is embedded in the canonical *COI1*-*JAZ* jasmonate signaling module. In the absence of *JA-Ile*, JASMONATE ZIM-DOMAIN (*JAZ*) repressors interact with MYC TFs and constrain their activity. Upon JA perception, the F-box receptor *COI1* promotes *JAZ* degradation, releasing MYCs to activate downstream defense genes [56,57,58]. A recent study indicates that the mentioned core module is reinforced by additional regulatory loops. In tomato (*Solanum lycopersicum*), *MYC2* transcriptionally activates *PUB22*, encoding a U-box E3 ubiquitin ligase that targets *JAZ4* for 26S proteasome-mediated degradation [59]. The *MYC2*-*PUB22*-*JAZ4* module enhances resistance to the insect *Helicoverpa armigera*. It also affects defense against the fungus *B. cinerea*, root growth inhibition, and anthocyanin accumulation, indicating that MYC-centered signaling is integrated with broader JA-dependent developmental and immune outputs [59].

MYC proteins also operate within higher-order transcriptional complexes. In *Arabidopsis* floral defense, *MED25*-based MYC-MYB complexes involving *MYC2/3/4/5* and *MYB28/29/76* mediate resistance to insects such as *H. armigera*, *Spodoptera exigua*, and *S. frugiperda* [60]. However, flower-specific bHLH factors *AMS* and *DYT1*, together with *MYB21/24*, antagonize these *MED25*-MYC-MYB complexes and suppress floral defense, revealing a tissue-specific regulatory layer that modulates JA immunity [60]. Similarly, rice NF-YA TFs physically interact with *OsMYC2/3* and interfere with the *OsMYC2/3-OsMED25* complex, thereby suppressing JA-mediated antiviral defense and facilitating infection by Rice stripe virus and Southern rice black-streaked dwarf virus [61].

The defense outputs controlled by MYC TFs are largely mediated through metabolic reprogramming. In *Arabidopsis*, *MYC3* and *MYC4* are major regulators of JA-induced tryptophan metabolism, while specific *JAZ* groups define the accumulation of aromatic amino acid-derived defense compounds and resistance to necrotrophic pathogens [62].

Guo et al. [54] further showed that the *JAZ*-MYC module coordinates tryptophan supply, indole glucosinolate production, and endoplasmic-reticulum body proliferation, linking transcriptional regulation to specialized defense biochemistry.

The experimental evidence supporting the functional centrality of bHLH/MYC TFs in plant defense is exceptionally strong. Their activity is shaped by repressor turnover, co-regulatory complexes, tissue-specific antagonism, and metabolic reprogramming. Current evidence strongly supports their role as immune hubs, although the functional diversification of MYC modules across species, tissues, and stress combinations remains an important frontier for future research.

#### 2.1.5. NAC Transcription Factors

Over the last five years, NAC TFs, with 101 studies identified, ranked as the fifth most extensively investigated TF group in the context of plant defense (Figure 1; Table 1). The mechanistic basis of NAC activity in immunity has recently been elucidated through the regulation of phytohormone pathways. Multiple NAC members function as direct transcriptional activators of SA biosynthesis [63]. *NbNAC1* in *N. benthamiana* enhances resistance to tobacco mosaic virus by directly binding a GAAATT motif in the promoter of *ICS1* (*isochorismate synthase 1*), the key SA biosynthetic gene. Silencing of *NbNAC1* suppresses SA signaling and systemic resistance, while its overexpression has the opposite effect [63]. JA signaling is equally subject to NAC regulation. In the tea plant (*Camellia sinensis*), *CsNAC2* cooperates with the MYB factor *CsMYB306* to activate *CsLOX2*, an early JA biosynthesis gene. This activation amplifies JA production in response to the herbivore-induced volatile (Z)-3-hexenyl acetate. Simultaneous suppression of both TFs additively attenuates herbivore (*Ectropis obliqua*) resistance [64]. A parallel module in the same plant species, involving *CsNAC30* and a TCP factor, directly activates a *BAHD acyltransferase* gene for the biosynthesis of the defensive volatile itself, placing NAC factors at multiple points within a single defense pathway [65].

A recurrent and notable feature of NAC biology is the use of membrane-associated isoforms as a regulatory switch. Under non-stress conditions, certain NAC proteins are retained at the endoplasmic reticulum membrane through a C-terminal transmembrane domain. Upon pathogen perception, they undergo regulated intramembrane proteolysis or signal-induced translocation, releasing the NAC domain to the nucleus. In Arabidopsis, NAC089 provides a clear example. Infection signals from *Phytophthora capsici* culture filtrate trigger *BAK1*-dependent translocation of *NAC089* from the endoplasmic reticulum to the nucleus via the Golgi apparatus. In the nucleus, NAC089 activates immune gene expression and contributes to resistance against both the oomycete *P. capsici* and the bacterium *P. syringae* [66]. Analogously, a truncated form of *BjuNAC62* lacking its transmembrane domain, mimicking the released nuclear-active form, confers tolerance to the fungus *Alternaria brassicicola* in Indian mustard (*Brassica juncea*) when induced and activates the defense-hormone signaling pathways [67].

NAC TFs are also embedded in networks involving negative regulatory NAC modules that prevent immune overactivation. In *Arabidopsis*, a triad of *NAC90*, *NAC61*, and *NAC36* (all induced by *P. syringae* pv. *maculicola*, SA, and the immune signal N-hydroxy pipecolic acid) acts as a coherent repressor complex. This complex directly suppresses expression of *NHP* and SA biosynthetic genes, including *ALD1* and *FMO1*, thereby preventing runaway immune amplification. However, nac90 knockout plants display constitutive immune activation and early senescence, illustrating the fitness cost of unrestrained NAC repression [68].

These findings reveal that NAC TFs act as multifunctional regulators of plant immunity, operating at distinct levels: activation of phytohormone pathways, spatial control through membrane-anchored isoforms, and fine-tuned modulation by repressor complexes. Such versatility positions NACs as central regulatory hubs within defense networks, balancing effective resistance to pathogens and herbivores while preventing the fitness and physiological costs associated with immune overactivation.

### 2.2. Emerging Frontiers in Plant Defense: Underexplored Transcription Factor Families

This section aims to synthesize relevant knowledge to broaden the spectrum of molecular actors implicated in plant resistance mechanisms. The TFs discussed in the following sections were not even among the ten least-cited TFs in the previously analyzed corpus retrieved from the PubMed database (Figure 1), highlighting the comparatively limited attention they have received over the last quinquennium. However, emerging evidence from the scientific literature has increasingly highlighted the important roles of the TFs discussed below in the plant defense context, thereby contributing to a more comprehensive understanding of the mechanisms through which they contribute to plant immunity. Owing to the more limited body of available evidence for these molecular actors, the literature was searched without temporal restriction, thereby allowing for a more comprehensive overview of the currently accessible literature.

#### 2.2.1. NF-Y Transcription Factors

The NF-Y (Nuclear Factor Y) TFs form heterotrimeric complexes composed of NF-YA, NF-YB, and NF-YC subunits, which recognize the DNA motif “CCAAT” in target promoters [69,70,71,72,73]. The NF-Y TFs are present in all eukaryotes. In plants, however, they have multiple independent NF-YA, NF-YB, and NF-YC subunits encoded by multiple genes. This organization creates an expanded repertoire compared to animals and fungi. It also allows the formation of diverse NF-Y trimer combinations that are frequently specialized in different tissues, developmental stages, and environmental conditions [71,74,75,76,77,78]. The NF-YB and NF-YC subunits contain histone-fold domains similar to histones H2B and H2A, respectively. They form a dimer that associates with NF-YA. NF-YA is responsible for most motif recognition, modulation of chromatin accessibility, and recruitment of other transcriptional regulators [70,73,75,79].

Functionally, plant NF-Y subunits are involved in multiple processes, including flowering, embryogenesis, root development, stress responses, and signal integration from different phytohormones [73,75,77,80,81]. Expression studies and promoter analyses indicate that many NF-Y genes contain cis-elements responsive to SA, JA, ET, and other stress signals. These findings reinforce the role of this TF family as an integrating axis between development and responses to abiotic and biotic stresses [72,76,77,78,82,83].

In the context of biotic stresses, different NF-Y subfamilies are directly related to plant-microorganism interactions in both beneficial and pathogenic associations [70,72,77]. Recent studies in rice have shown that certain NF-Y molecules act as negative regulators of JA-mediated immunity, repressing antiviral defense genes and favoring viral infection [61]. Thus, repression of these NF-YA factors leads to increased resistance [61,77].

In addition, genomic and transcriptomic analyses in *Arabidopsis* [72], potato (*Solanum tuberosum*) [84], citrus (*Citrus* spp.) [85], and poplar (*P. davidiana × P. bollena*) [86] revealed that NF-Y genes are differentially expressed after infection by fungi, bacteria, or viruses. These genes also cluster into enriched co-expressed modules associated with defense genes [73,77,82,87]. In *Arabidopsis*, for example, characterization of the NF-YB subfamily revealed expression profiles induced by biotic and abiotic stresses. The analysis also identified promoters associated with hormones and pathogen responses. These findings suggest that different combinations of NF-YB, NF-YA, and NF-YC regulate immunity according to the pathogen and physiological context [72,82,88]. For example, the overexpression of NF-Y genes in *Arabidopsis* and soybean resulted in increased tolerance to viruses, bacteria, fungi, and SCN [88]. On the other hand, the overexpression of the NF-Y complex (*MeNF-YA1*/3, *MeNF-YB11*/16, and *MeNF-YC11*/12) in cassava (*Manihot esculenta*) resulted in decreased disease resistance to the bacterial pathogen *Xanthomonas axonopodis* pv. *Manihotis* by activating pathogenesis-related promoters [89].

These data reinforce the view that NF-Y TFs are regulatory hubs in different layers of the plant immune response, contributing to chromatin regulation, hormonal signaling, and transcriptional reprogramming during development and responses to biotic stresses [70,72,73,76,77,82].

#### 2.2.2. Trihelix Transcription Factors

Trihelix TFs (also known as GT TFs) comprise a plant-specific gene family with a “trihelix” DNA-binding domain characterized by three α-helices arranged in a helix-loop-helix-loop-helix structure [90,91]. These TFs recognize GT-rich cis-elements such as GT-1/GT-2 [GGTTAA/GTTAC] motifs, originally described in light-responsive promoters [90,92]. The trihelix domain contains conserved basic residues that interact with the major groove of DNA and hydrophobic residues that stabilize helix packing. At the same time, the C-terminal regions and intrinsically disordered segments act as transcriptional regulatory modules or protein-protein binding interfaces [90,91,93].

Functionally, Trihelix TFs are recognized as multifunctional regulators of development (e.g., embryogenesis, flowering, and seed formation), integrators of hormonal signals mediated by ET and abscisic acid (ABA), and regulators of adaptation to stress in plants [90,92,94,95,96,97]. These functions are evidenced in species such as *Arabidopsis* [92], tomato [96], pine (*Pinus massoniana*) [98], cucumber (*Cucumis sativus*) [99], maize (*Zea mays*) [100], and pea (*Pisum sativum*) [101]. In these species, promoters of Trihelix genes are enriched for responses to ABA, SA, JA, auxin, and gibberellin. This pattern reinforces their role as integrators of hormonal and environmental signals [91,94,98,102].

In the context of biotic stress, accumulating evidence indicates that Trihelix TFs are directly involved in plant-pathogen interactions and immune signaling. In grapevine (*Vitis vinifera*), for instance, members of this family have been shown to play important roles in resistance to anthracnose [93]. In the model plant *Arabidopsis*, members of the SIP1 subfamily have been implicated in *Agrobacterium*-mediated transformation, modulating host susceptibility to crown gall tumor formation [90,92].

Trihelix activity has also been implicated in the regulation of PTI and ETI in *Arabidopsis*, where the *ASR3* factor, related to the SH4 subfamily, acts as a negative regulator of PTI by attenuating the immune response [92,103]. When *ASR3* interacts with another Trihelix TF, designated *ASR3-Interacting Transcription Factor 1*, these proteins can form both heterodimers and homodimers in planta, thereby enhancing resistance to *P. syringae* [92]. Conversely, double-knockout mutants exhibit reduced resistance, suggesting that *ASR3* and its interacting partner act in an interdependent manner to regulate immune gene expression and defense responses [92]. Another Trihelix TF, *GT-3a*, is specifically induced during ETI and acts as a negative regulator of bacterial resistance by repressing SA-dependent defense genes in *Arabidopsis* [92,104]. Also in *Arabidopsis*, the Trihelix TF *GT-2-like 1* is a substrate of *MPK4*, positively regulates defense-related gene expression, and inhibits factors that mediate growth and development [105].

Omics analyses of pine subjected to pinewood nematode (PWN) infection revealed that PmTrihelix *PmGT9*, *PmGT35*, and *PmGT54* responded to SA treatment. In contrast, *PmGT14*, *PmGT26*, and *PmGT54* showed significant responses to methyl-JA, indicating their potential roles in PWN resistance through hormonal signaling pathways [98]. Similar findings were obtained in cucumber, reinforcing the participation of Trihelix TFs in transcriptional defense networks: CsTrihelix genes that were differentially expressed after fungal infections (cucumber scab, *Cladosporium cucumerinum*, and gray mold, *B. cinerea*) clustered into co-expressed modules enriched in defense pathways, phenylpropanoid biosynthesis, and hormonal signaling. In some of these modules, Trihelix TFs emerge as hubs, showing connectivity with pathogenesis-related protein receptor kinases (e.g., LRR-RLKs), pointing to a coordinating role in defense gene reprogramming during pathogen recognition [99].

In functional assays, overexpression or silencing of individual Trihelix genes significantly altered resistance to bacterial or fungal pathogens, followed by changes in SA/JA marker expression [92,98,104]. Thus, Trihelix TFs operate as key regulatory hubs within hormone-dependent immune networks, modulating the defense response and shaping the balance between growth and immunity in plants [92,103,104].

#### 2.2.3. PLATZ Transcription Factors

The PLATZ TFs constitute a plant-specific family that possesses an AT-rich promoter sequence-binding domain and a zinc motif, which confer structural stability and the ability to interact with other regulators [106,107]. Initially described in seed development and vegetative growth processes, members of this group have been associated with stress responses, acting as integrators of hormonal signaling, energy metabolism, and stress adaptation [108,109].

Under biotic stresses, genomic and transcriptomic analyses in cucurbits have shown that different PLATZ genes were induced following infection by powdery mildew and other foliar and root fungal pathogens. Among them, *CpPLATZ4* (in *Cucurbita pepo*) and *ClPLATZ1*, 3, 4, and 6–10 (in *Citrullus lanatus* L.) have been identified as positive regulators of disease resistance, modulating defense signaling pathways and maintaining cell wall integrity [107,110]. Consistently, co-expression analyses revealed that PLATZ genes cluster within defense-associated regulatory modules and participate in transcriptional reprogramming during infection [109,110,111,112]. Taken together, these data point to PLATZ TFs as emerging regulators in defense networks, integrating environmental, hormonal, and pathogen-derived signals to coordinate defense responses [107,108,109,112].

#### 2.2.4. TCP Transcription Factors

The TCP TF family brings together plant-specific TFs that possess a basic helix-loop-helix domain capable of recognizing promoters with GC-rich motifs [113,114,115]. Beyond their classic role in developmental control, TCPs have been identified as connectors between growth and immunity. Members of classes I and II modulate the JA pathway and interact with central components of immunity. Through these interactions, they affect both the expression of defense genes and cell cycle processes [114,116,117,118,119]. Class I TCPs (such as *TCP8*, *TCP14*, and *TCP15*) form complexes with *NPR1* to activate PR genes in the SA pathway. TCP14 and TCP15 also associate with MOS1 to adjust *SNC1* expression and genes linked to endoreduplication. This activity integrates immune signaling (including crosstalk with JA) with growth control [117,118]. ChIP-Seq and RNA-Seq analyses revealed that TCPs control the enrichment of gene sets in hormonal and defense signaling pathways, positioning them as hubs in networks that integrate development and immune responses [119]. Thus, TCPs are recognized as important TFs in plant immunity, acting as direct modulators of defense gene expression [113,114,117,118].

#### 2.2.5. GRAS Transcription Factors

The GRAS TF family comprises a group of nuclear regulators that act as coactivators or corepressors to integrate hormonal signals and participate in growth, development, and stress responses [120,121,122]. Structurally, GRAS proteins contain a highly conserved C-terminal domain characterized by typical motifs, including LHRI, VHIID, LHRII, PFYRE, and SAW, which are involved in protein-protein interactions and transcriptional regulation. In contrast, their variable N-terminal region is frequently associated with functional specificity and the integration of distinct signaling pathways [120,122,123]. Functionally, several GRAS subfamilies, such as DELLA, SHR/SCR, PAT1, and LISCL, regulate processes ranging from root architecture and meristem development to arbuscular mycorrhizal formation and stress responses, thereby coordinating multiple hormonal pathways [121,122,124,125].

In the context of biotic stress, members of the GRAS family have been associated with pathogen resistance by modulating gibberellin (GA), SA, and JA signaling pathways [126,127,128,129,130]. Transcriptomic analyses in apple have shown that several SCL- and DELLA-type genes are induced by necrotrophic fungal pathogens, whereas GA-responsive genes are concomitantly repressed, suggesting a GRAS-mediated metabolic redirection toward defense responses [121,130]. Patterns of tissue-dependent regulation have been observed in grapevine, in which the GRAS gene *VviSCL3b* is expressed across multiple tissues, including stems, seeds, leaves, buds, and berries, and is upregulated upon infection by the fungus *B. cinerea* [129]. Conversely, *VviLAS2* expression decreases after *B. cinerea* infection, although it is co-expressed with genes potentially involved in biotic stress responses [129]. In rice, functional analyses have identified the SCL subfamily member *OsSCL7* as a positive regulator of immunity against the foliar pathogen *M. oryzae*. *OsSCL7* contributes to disease resistance mainly through the transcriptional regulation of defense-related genes, further supporting the involvement of GRAS proteins in plant immune responses [120,131].

Omics-based analyses in wheat (*Triticum aestivum*), birch (*Betula platyphylla*), Chinese tulip tree (*Liriodendron chinense*), and other species have revealed that GRAS genes cluster into co-expression modules under combined stress conditions. These modules are frequently enriched for defense-related genes, hormonal signaling components (including GA, SA, ABA, and methyl jasmonate, MeJA), and genes responsive to external stimuli. Within these networks, members of the PAT1, LISCL, and DELLA subfamilies often emerge as potential regulatory hubs [122,125,129,132,133,134]. In this context, GRAS TFs can be regarded as emerging yet underexplored regulators of plant immunity, acting as molecular bridges between development, hormonal signaling, and defense networks. Their regulatory versatility highlights their potential utility in breeding and genome-editing strategies to improve disease resistance [120,121,125,129,134].

#### 2.2.6. Research Potential and Future Priorities Among Emerging TF Families

Among the emerging TF families discussed here, NF-Y and TCP currently appear to offer the strongest near-term research potential, although for partly different reasons. The research potential of NF-Y complexes reflects their combinatorial organization and their involvement in chromatin regulation [70,73,75,79], hormonal signaling [72,76,77,78,82,83], and both positive and negative immune responses [61,88]. Together, these features make individual NF-Y subunits and specific heterotrimeric assemblies attractive targets for elucidating context-dependent defense mechanisms and for precision breeding or genome-editing approaches. TCP factors are similarly promising because genetic and molecular evidence directly places them at the interface between growth and immunity, linking SA–JA crosstalk, immune-receptor expression, and cell-cycle regulation [114,116,117,118,119]. PLATZ TFs, in turn, may represent the family with the greatest discovery potential because their involvement in disease resistance has only recently begun to receive functional support, leaving their upstream regulators, direct DNA targets, and possible roles in cell-wall-associated defense largely unresolved [107,108,109,110,111,112].

## 3. How Are the Regulators Regulated?

TFs can be regarded as key molecular “spearheads” of the plant response to pathogens. By activating or repressing specific target genes, they drive cellular reprogramming and enable plant cells to adjust more effectively to the newly imposed stress condition. This observation raises a central question: how are these molecular “spearheads” themselves regulated to remain properly activated, coordinated, and functionally competent to perform their vital roles?

To achieve the requisite plasticity in immune signaling, plants employ a hierarchical control system that targets TFs at every stage of their biogenesis and function. This section discusses how such molecular actors are modulated by transcriptional and post-transcriptional filters, as well as post-translational switches. These integrated layers enable activation and precise attenuation of defense programs necessary to minimize fitness costs [19,135,136].

### 3.1. Transcriptional Regulation

Among the best-characterized examples of transcriptional control of TFs associated with plant defense is the autoregulatory and hierarchical network governing WRKYs. Several WRKY genes contain W-box elements in their own promoters, making them direct transcriptional targets of other WRKY proteins and thereby creating a feed-forward and feedback architecture that shapes the amplitude and duration of the defense transcriptome [137,138]. In *Arabidopsis*, *WRKY33* provides a clear example of WRKY-to-WRKY transcriptional regulation during pathogen attack. Upon *B. cinerea* infection, *WRKY33* binds the promoters of multiple WRKY genes, including its own promoter. It modulates the expression of *WRKY38*, *WRKY41*, *WRKY48*, *WRKY50*, *WRKY53*, and *WRKY55*, supporting extensive auto- and cross-regulation within the WRKY defense network [139]. Likewise, hierarchical WRKY regulatory networks are present in rice. For example, *OsWRKY6* directly activates *OsWRKY45* and *OsWRKY47* [140], indicating that WRKY-based transcriptional cascades extend beyond *Arabidopsis*.

Chromatin-level regulation further modulates TF gene transcription, as several H3K4 methyltransferases are implicated in the regulation of plant immunity. For example, *ATX1* acts as a “master regulator” in activating *WRKY70* expression, a mediator of crosstalk between the SA- and JA-signaling pathways, by promoting H3K4me3 deposition [141]. Notably, Cai et al. [142] showed that the chromatin remodeling complex SWR1 enhances resistance to the white mold fungus *S. sclerotiorum* in *A. thaliana* through a process mediated by receptor-like kinase *ERECTA* (*ER*) signaling. The authors identified a series of *WRKY33* target *YODA DOWNSTREAM* (*YDD*) genes. They further demonstrated that SWR1 and *ER* signaling are required to enrich the H2A.Z histone variant and the H3K4me3 histone modification at YDDs. These pathways also promote WRKY33 binding to YDD promoters upon *S. sclerotiorum* infection. In turn, HDA19, an *Arabidopsis* histone deacetylase, negatively regulates the transcription of the defense-associated TFs *WRKY38* and *WRKY62*, two negative regulators of plant basal defense, by maintaining a repressive chromatin state at their promoters in the absence of pathogen stimuli [143].

### 3.2. Post-Transcriptional Regulation

Beyond transcriptional control, post-transcriptional regulation by non-coding RNAs targeting defense-related TFs represents a critical regulatory layer essential for fine-tuning the immune response, enabling rapid responses (within minutes) that transcriptional regulation alone (in hours) cannot achieve [144,145].

MicroRNAs (miRNAs) of 21–24 nucleotides act as highly specific negative regulators of immunity transcripts, keeping defense-related TFs and pattern-recognition components in check under basal conditions and relieving this brake upon pathogen perception [146]. For instance, *miR393* negatively regulates *TIR1*/*AFB* auxin receptors during PTI, redirecting metabolic resources toward defense [144,145,146], while *miR160* targets *ARF10*/16/17 transcripts, modulating the growth-defense balance [147].

Long non-coding RNAs (lncRNAs) exceeding 200 nucleotides regulate target gene expression via cis- or trans-acting mechanisms, functioning primarily through complex, three-dimensional structures [144,145,148]. The lncRNA *ELENA1* is a PAMP-induced molecular scaffold essential for PR1-mediated resistance in *A. thaliana* [145,149]. It provides interaction surfaces that increase the local concentration of partners, including *NPR1* and chromatin-modification complexes, to facilitate the assembly of transcriptional complexes at defense gene promoters [144,145,146,150]. Cis-acting lncRNAs modulate the transcription of genes in close genomic proximity by recruiting, stabilizing, or displacing TFs [151]. In *A. thaliana*, the lncRNA *SABC1* limits SA-mediated defense under basal conditions by repressing the neighboring TF *NAC3* through Polycomb-mediated chromatin silencing [151]. This mechanism reduces *ICS1* (*isochorismate synthase 1*) expression and SA biosynthesis [151]. Pathogen infection decreases *SABC1* expression, enabling *NAC3*- and *ICS1*-dependent activation of antibacterial and antiviral defenses [151]. Transcriptome analyses indicated that Turnip crinkle virus (TCV) infection in *A. thaliana* represses the expression of the APETALA2 (AP2) TF through the action of lncRNA *LINC-AP2* [152]. *LINC-AP2* overexpression reduces AP2 expression and increases TCV coat protein accumulation, thereby connecting lncRNA activity to TF regulation and viral susceptibility [152].

LncRNAs also participate in tomato defense against *Phytophthora infestans* by acting as competing endogenous RNAs (ceRNAs) to modulate microRNA (miRNA) activity. Transcriptomic and degradome sequencing data revealed that specific lncRNAs act as miRNAs targeting defense-related TFs, exemplified by the *lncRNA42705/lncRNA08711-miR159*-MYB module [153]. Following *P. infestans* infection, *lncRNA42705* and *lncRNA08711* expression levels correlate negatively with *miR159* and positively with the expression of MYB genes [153]. Silencing these lncRNAs increases *miR159* levels and reduces MYB expression, confirming their regulatory control over TF expression during pathogen interactions [153].

### 3.3. Post-Translational Regulation

Converging on synthesized defense TFs, post-translational modifications (PTMs) operate as a multidimensional regulatory code that decodes pathogen-derived signals, PAMP perception, effector recognition, and hormonal cues into context-specific changes in TF activity, fundamentally altering the protein’s physicochemical properties and activity status [154,155].

#### 3.3.1. MAPK Cascades and TF Phosphorylation

In plant immunity, MAPK (Mitogen-Activated Protein Kinase) cascades operate as tripartite (MAPKKK > MAPKK > MAPK) modules that amplify pathogen-perception signals from cell-surface PRRs and intracellular NLRs through sequential phosphorylation. Specificity within this cascade is ensured not only by substrate sequence but by docking domains [156,157]. MAPKs (such as *MPK3*/6) possess a d-site that recognizes short linear motifs (SLiMs) in their substrates (such as *WRKY33*), allowing for low-affinity, high-specificity interactions essential for catalytic phosphorylation [158,159,160].

The covalent addition of a phosphate group (PO_4_^3−^) is a drastic event: it introduces a bulky, highly negatively charged group where a neutral hydroxyl group (-OH) previously existed. The impact is immediate. First, electrostatic allostery occurs when the new charge forces a conformational change by repelling acidic residues or attracting basic ones. This change alters DNA affinity [135,150,161]. Second, phosphorylation can create docking sites for 14-3-3 domains, which possess a positively charged pocket [150,157,161]. For example, WRKY33 phosphorylated by MPK3/MPK6 at residues S55, T105, and S176 exhibits increased affinity for W-boxes [157,162].

In *A. thaliana*, the primary defense cascade (MEKK1 > MKK4/MKK5 > MPK3/MPK6) is activated within minutes of PAMP perception [158,159,160]. Acting at the apex of these cascades, MPK3/MPK6 phosphorylate WRKY33 cooperatively, with multi-site phosphorylation requiring both MAPKs simultaneously [158,159]. This phosphorylation is essential for WRKY33 activity and subsequent biosynthesis of camalexin, a known antimicrobial compound [159]. In parallel, MPK4 phosphorylates MKS1, releasing WRKY33 from inhibitory *MKS1*-*WRKY33* complexes [159,160], exemplifying regulation via molecular sequestration. Expanding targets beyond WRKYs, MPKs also phosphorylate the ERF6 TF, stabilizing it and promoting inhibitory responses to necrotrophic pathogens [158,159,160].

#### 3.3.2. CDPKs and Calcium Signal Transduction

Converging on common substrates of the MAPKs but responding to distinct cues, CDPKs (Calcium-Dependent Protein Kinases) detect the cytosolic Ca^2+^ oscillations triggered by PAMP perception and effector-induced resistosome activation [163,164]. They translate this defense-specific ionic signature into enzymatic activity via EF-hand domains [164,165]. These are helix-loop-helix structural motifs in which the loop is rich in acidic residues (Asp, Glu), forming a negatively charged binding pocket. The influx of the divalent cation Ca^2+^ cooperatively saturates these sites, neutralizing charges and inducing a drastic conformational change that releases the kinase domain from autoinhibition [163,164,165,166]. This mechanism links temporal calcium signatures to appropriate transcriptional responses. *CPK5*/*CPK6* exemplify this specificity by phosphorylating WRKY8 and WRKY28 with antagonistic effects: phospho-WRKY8 promotes immunity, whereas phospho-WRKY28 suppresses it [167,168]. Additionally, CPK28 phosphorylates the bZIP TF TGA3, potentiating its activity and integrating calcium signals with the SA pathway [167,168].

#### 3.3.3. PP2C Phosphatases and Ubiquitin Ligases as Attenuators

Establishing the equilibrium necessary to prevent autoimmunity, PP2C phosphatases antagonize the MAPK cascades described above by removing phosphate groups and thereby acting as negative-feedback and deactivation mechanisms that terminate defense signaling once the threat is cleared [169]. Clade “A” PP2Cs also regulate immunity [170]; PP2C5 dephosphorylates MPK3/MPK6, attenuating PTI; while PP2C38 interacts with the FLS2-BAK1 receptor complex, uncoupling it after activation to prevent deleterious hyperimmunity [169,170,171]. The functional importance of these phosphatases is underscored by the phenotype of the *PP2C5-PP2C38* double mutant, which exhibits constitutive activation of defenses and severe dwarfism [171].

Complementing phosphorylation control, the ubiquitin-proteasome system governs TF turnover. RING ligases such as MIEL1 ubiquitinate MYB30, promoting its degradation [172]. More elaborately, the SCF-COI1 complex exemplifies ligand-regulated degradation, functioning via a molecular glue mechanism. The interaction between COI1 (F-box) and the JAZ repressor is thermodynamically unstable. The hormone *JA-Ile*, a hydrophobic amino acid conjugate, acts as the glue, inserting into pockets in both proteins and stabilizing a ternary complex (COI1-[JA-Ile]-JAZ) [58]. This capture leads to K48-ubiquitination of *JAZ* and its rapid degradation, releasing MYC2/MYC3/MYC4 [173]. The competition between stabilizing sumoylation and degradative ubiquitination on the same lysine creates a bistable molecular switch. This complex network of enzymatic regulation is the central battleground in the coevolution between plants and pathogens [58,174].

### 3.4. Crosstalk Between TFs and Hormone Receptors

Integration of the defense hormone network—SA against biotrophs and JA and ET against necrotrophs and chewing herbivores—constitutes the highest level of regulation, allowing plants to tailor their response to the pathogen’s lifestyle [136,155,175,176]. Defense TFs, such as NPR1-recruited TGAs and JA-responsive MYC2, function as the integrating nodes where these hormonal inputs converge into a single transcriptional output [176].

#### 3.4.1. Salicylic Acid Pathway: NPR1-TGA

SA signals through the master regulator *NPR1* [177]. Under basal conditions (low SA), NPR1 exists as a cytoplasmic oligomer maintained by covalent disulfide bridges (-S-S-) between cysteine residues (C82, C216) [150]. SA accumulation induces a cellular redox change (via thioredoxins, TRXh5) that catalyzes the reduction of these bridges to thiol groups (-SH-HS-) [177,178]. This constitutes a redox switch: bond cleavage dismantles the oligomer, exposing basic residue-rich regions recognized by importins [177]. Once in the nucleus, NPR1 functions as a coactivator, interacting via its BTB/POZ domain with TGA TFs (bZIP family) [150,177]. The NPR1-TGA complex subsequently recruits HAC1, which acetylates H3K9/K14 and opens chromatin for PR gene transcription [177,178]. When additional signals are integrated, TGA3 phosphorylation by BIN2 (activated by SA) increases its DNA affinity [179].

#### 3.4.2. Jasmonic Acid Pathway: COI1-JAZ-MYC2

Operating frequently in antagonism to SA, JA and its active conjugate JA-Ile mediate defense against necrotrophs [58,174]. In the absence of JA-Ile, JAZ repressors (containing Jas and EAR domains) bind to the TPL corepressor (via NINJA) while simultaneously sequestering activator TFs such as MYC2, blocking transcription [180,181,182]. The accumulation of JA-Ile stabilizes the tripartite JAZ-COI1-JA-Ile interaction (a molecular glue mechanism), promoting JAZ degradation via SCF-*COI1* [180,181,182]. This releases MYC2, which then dimerizes (via its bHLH domain) and binds to E-boxes (CACGTG), activating genes such as *VSP2* and *PDF1.2* [183,184]. Notably, MYC2 transcriptionally activates *JAZ* genes, establishing a negative feedback loop that terminates signaling [183,184].

#### 3.4.3. Salicylic Acid—Jasmonic Acid Antagonism

Connecting these pathways, SA-JA antagonism is mediated by direct molecular competition [181]. TGA TFs act as integration points: they can interact with both NPR1 (to activate the SA pathway) and JAZ repressors (to repress the JA pathway) [177]. The result is a competitive equilibrium dictated by the relative concentrations of regulators [181]. NPR1 also mediates antagonism by sequestering TGA TFs required for JA gene expression [177,185]. This antagonism has co-evolutionary implications: effectors such as HopZ1a that stabilize JAZs (suppressing JA) inadvertently potentiate SA responses, suggesting that biotrophic pathogens have evolved to suppress JA. In contrast, necrotrophs suppress SA [186,187]. This trade-off allows for the prioritization of defenses, optimizing metabolic costs and defensive efficacy [181,186,187].

## 4. Cell-Type-Specific Transcription Factor Activity in Plant Immunity

Historically, plant immunity has been interpreted as a coordinated and relatively uniform response at the tissue level, largely owing to the predominance of transcriptomic approaches based on homogenized tissues, particularly bulk RNA sequencing (bulk RNA-Seq). Although these strategies have been instrumental in identifying the core pathways underlying plant defense against pathogens, they impose important conceptual limitations. Plant immune responses emerge from the interplay among distinct cell types, the spatial organization of host tissues, and the distribution of invading pathogens. Consequently, plant immunity emerges as a dynamic mosaic of defense-activated and infection-permissive cells, a degree of heterogeneity that conventional bulk transcriptomic approaches are unable to resolve [188]. In this context, bulk transcriptomic analyses dilute the transcriptional signatures of specific cellular subpopulations, thereby obscuring precisely those responses that depend on cell-to-cell interactions with the pathogen.

The advent of cell-resolution technologies, such as single-cell RNA sequencing (scRNA-Seq) [189] and spatial transcriptomics [190], has driven a profound paradigm shift by revealing that plant tissues are composed of a dynamic mosaic of functionally distinct cellular states [188,191,192]. A recent study by Song et al. [188] on *A. thaliana* integrating both approaches demonstrated that even seemingly homogeneous tissues exhibit remarkable transcriptional diversity, reflecting immune programs that are tightly compartmentalized according to cell type. Plant immunity operates through cell-type-specific mechanisms [188]. Pavement cells activate SA biosynthesis via *ICS1* and *EDS5*, while guard cells remain transcriptionally silent for this pathway, instead mounting autonomous responses involving Ca^2+^ influx, reactive oxygen species (ROS) accumulation, and hypersensitive cell death [188]. This functional compartmentalization, conserved across plant-fungal and plant-bacterial interactions, reframes plant immunity as an emergent phenomenon arising from spatiotemporal integration of heterogeneous cellular states [188].

At the core of this spatiotemporal organization lies TF activity. The pioneering study by Bai et al. [193] on woodland strawberry (*Fragaria vesca*) infected with *B. cinerea* demonstrated that transcriptional activation is not uniform but rather a tightly compartmentalized process dependent on both cell type and infection stage. During the early incubation phase (6 hpi), TF families such as WRKY, NAC, TIFY, and HSP display highly tissue-specific expression signatures. WRKY75 is enriched in the upper epidermis, whereas HSP90 accumulates predominantly in hydathodes and vascular bundle sheath cells. ZAT11 is preferentially expressed in the phloem. As fungal colonization progresses (12 hpi), however, the transcriptional landscape transitions toward a more distributed configuration, in which TFs such as *WRKY75*, *HSF4*, and *ZAT11/12* become simultaneously expressed across multiple cellular lineages.

Using scRNA-Seq, Yan et al. [194] unraveled the cellular heterogeneity underlying maize responses to *Puccinia polysora* infection by constructing the first single-cell atlas of leaves from contrasting maize cultivars challenged with this fungal pathogen. Their analysis revealed that the resistant line (R99) maintains a pre-activated immune-like state even in the absence of pathogen exposure (mock treatment), a pattern that is particularly pronounced in guard and epidermal cells. Within these cell populations, TFs belonging to the WRKY, ZIM, MYB, and AP2-EREBP families exhibit constitutively elevated expression levels under mock conditions. Notably, AP2-EREBP emerged as a central regulatory hub co-activated in both guard and epidermal cells, integrating signaling pathways that confer resistance to biotic stresses while coordinating the transition from basal physiological programs to defense-associated states. This spatiotemporal organization suggests that resistance to *P. polysora* is not solely dependent on the inducible activation of downstream defense effectors but rather on the prior establishment and maintenance of cell-type-specific transcriptional programs [194]. Consequently, the regulatory architecture mediated by WRKY, ZIM, MYB, and AP2-EREBP TFs forms an early transcriptional barrier that restricts fungal colonization, highlighting the potential of breeding strategies to constitutively modulate key TFs in host-pathogen interface cells.

The cellular heterogeneity revealed during *A. thaliana* infection with *P. syringae* identified the TF *CBP60g* as a central regulator of plant immunity [195]. Predominantly expressed in immune-associated cell populations, *CBP60g* acts in concert with *SARD1* to regulate SA biosynthesis, a key pathway underlying defense against biotrophic pathogens. Spatial analyses further demonstrated that *CBP60g* expression occurs both in cells adjacent to bacterial colonization sites and throughout more distal regions of the leaf, suggesting a dual role in coordinating local defense activation and systemic immune signaling [195]. In addition, pseudotime analysis, which computationally orders cells along a trajectory representing disease progression, positioned immune-associated cell states at early stages of disease progression and identified *CBP60g* among the transcriptional regulators linked to these responses [195]. This temporal pattern indicates that *CBP60g* contributes to the early establishment of defense programs before the onset of susceptible cellular states, reinforcing its role in orchestrating the transcriptional reprogramming required for effective resistance to pathogen colonization [195].

The spatial organization of immune responses in *A. thaliana* leaves further revealed that distinct TF networks operate in specialized immune cell states [104]. Cells located at the center of immune-active hotspots, termed primary immune responder (PRIMER) cells, were characterized by the enrichment of CAMTA and GT-3A regulatory motifs. While CAMTA factors are known repressors of SA biosynthesis through the suppression of *CBP60g* and *SARD1*, the trihelix TF *GT-3A* emerged as a previously unrecognized regulator of leaf immunity. Nobori et al. [104] proposed that *GT-3A* may locally modulate SA signaling to balance defense activation and hypersensitive cell death during early pathogen recognition. In contrast, neighboring bystander cells exhibited enrichment of WRKY motifs and preferentially activated systemic defense-associated genes such as *ALD1* and *FMO1*. These findings support a model in which cell-state-specific TFs coordinate complementary defense programs: *CAMTAs* and *GT-3A* regulate localized responses in PRIMER cells, whereas WRKY-dependent networks promote immune signal propagation and systemic resistance in surrounding tissues [104].

The increasing application of single-cell, spatial, and multi-omics approaches has expanded our understanding of how TFs coordinate local and systemic immune responses across distinct cellular populations. Additional recent studies exploring the relationship between cellular dynamics and TF-mediated immune regulation are summarized in Table 2.

## 5. Effector-Mediated Reprogramming of Plant Immunity Through the Manipulation of Host Transcription Factors

As TFs operate as central regulators in plant defense, it is unsurprising that pathogens have evolved strategies to manipulate these molecules. The cell-type-specific (Section 4) and network-level importance of TFs (Section 7.1) make TFs attractive targets for pathogen effectors seeking to suppress immunity, redirect host metabolism, or create a cellular environment favorable to colonization.

**Figure 2:**
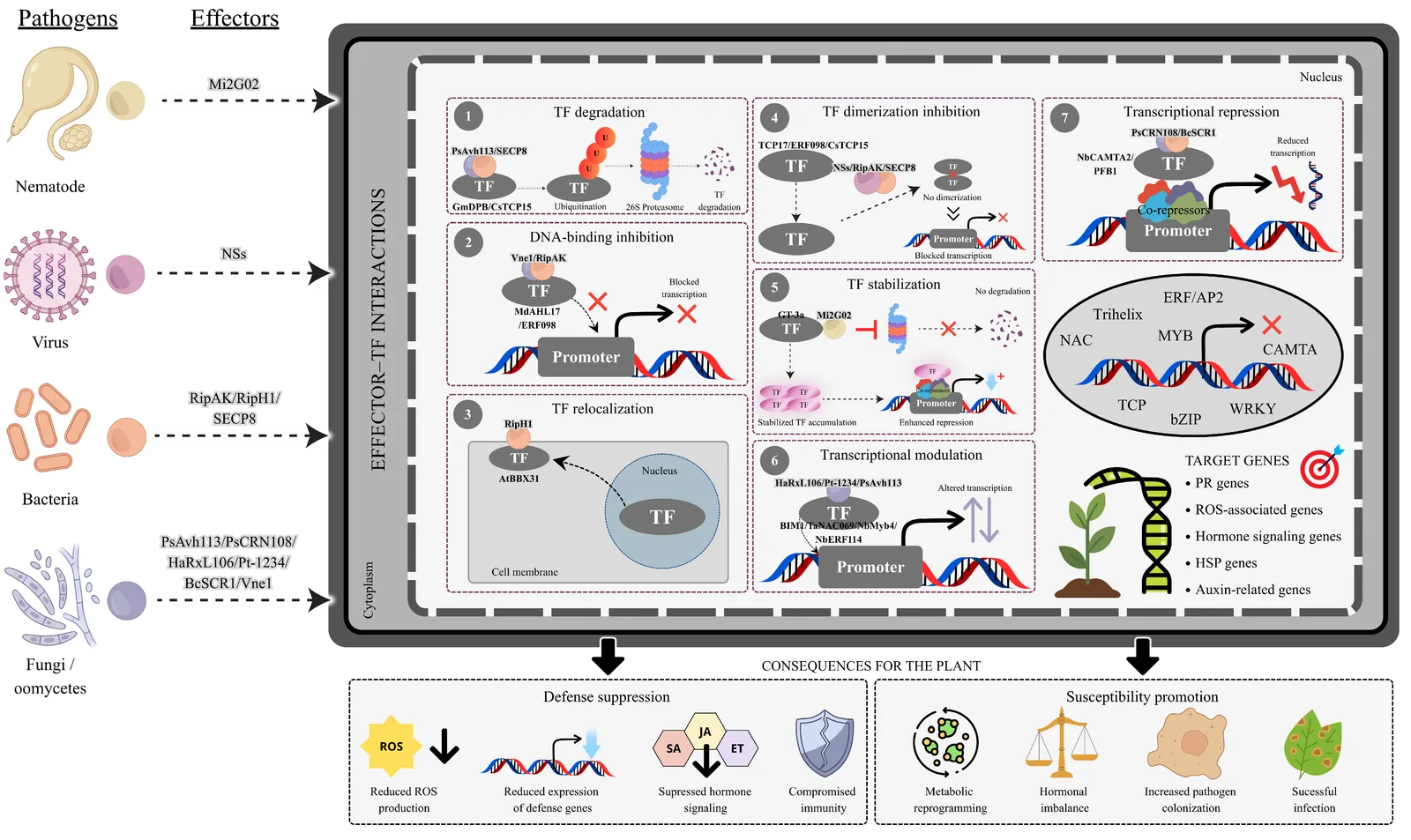
Schematic model of pathogen effector-mediated manipulation of plant transcription factors and its consequences for host immunity. Pathogen-secreted effectors from nematodes, viruses, bacteria, fungi, and oomycetes may target host transcription factors (TFs) through seven major, non-mutually exclusive mechanisms: (1) TF degradation through ubiquitination and the 26S proteasome pathway; (2) inhibition of TF binding to promoter regions; (3) TF relocation between the nucleus and cytoplasm; (4) inhibition of TF dimerization; (5) TF stabilization and accumulation; (6) modulation of TF-dependent transcription; and (7) recruitment or activation of transcriptional co-repressors. These mechanisms may prevent TFs from activating defense-related genes or, alternatively, enhance the activity of TFs that negatively regulate plant immunity. The representative effector–TF interactions shown in the figure derive from different plant–pathogen systems and illustrate alternative molecular strategies rather than sequential steps of a single signaling pathway. Effector-mediated interference with TF activity may reshape the expression of pathogenesis-related genes, reactive oxygen species-associated genes, hormone signaling genes, heat shock protein genes, and auxin-related genes. These transcriptional alterations may reduce the production of reactive oxygen species; suppress salicylic acid-, jasmonic acid-, and ethylene-dependent signaling; compromise immune responses; promote metabolic reprogramming and hormonal imbalance; and consequently favor pathogen colonization and successful infection. Abbreviations: PR genes, pathogenesis-related genes; ROS, reactive oxygen species; HSP, heat shock protein; SA, salicylic acid; JA, jasmonic acid; ET, ethylene; ERF/AP2, ethylene-responsive factor/APETALA2 transcription factor family; NAC, NAM/ATAF/CUC transcription factor family; MYB, myeloblastosis-related transcription factor family; TCP, TEOSINTE BRANCHED1/CYCLOIDEA/PROLIFERATING CELL FACTOR transcription factor family; WRKY, WRKY-domain transcription factor family; CAMTA, calmodulin-binding transcription activator; bZIP, basic leucine zipper transcription factor family; Trihelix, trihelix DNA-binding transcription factor family; U, ubiquitin; 26S proteasome, ubiquitin-dependent protein degradation machinery. Mi2G02, NSs, RipAK, RipH1, SECP8, PsAvh113, PsCRN108, HaRxL106, Pt-1234, BcSCR1, Vne1, PFB1, BIM1, TaNAC069, NbMyb4, NbERF114, NbCAMTA2, AtBBX31, GT-3a, MdAHL17, ERF098, TCP17, CsTCP15, and GmDPB designate specific pathogen effectors or host transcription factors included as representative examples in the model.

Pathogens may interfere with TF function directly by binding, modifying, degrading, or relocating specific TFs (Figure 2) or indirectly by altering upstream signaling pathways, chromatin states, hormone balance, or co-regulatory complexes. This molecular interference illustrates that the plant immune transcriptional landscape is not merely a host-controlled system but a contested regulatory arena shaped by host-pathogen coevolution. In this context, TFs occupy a strategic position: they are both defenders of plant cellular integrity and vulnerable points of pathogen exploitation. Examining how pathogens target TFs and transcriptional hubs, therefore, provides critical insight into the molecular logic of susceptibility, resistance breakdown, and the evolutionary pressure shaping immune regulatory networks.

As an example, *P. sojae* (Ps) deploys multiple effectors to modulate the activity of TFs across different plant species, thereby promoting broad transcriptional reprogramming in favor of pathogen colonization. The effector PsAvh113 (Figure 2) displays a multifaceted mode of action. In soybean, this molecule interacts with the GmDPB TF (Figure 2), promoting its proteasome-mediated degradation and reducing the expression of defense-associated genes such as *GmCAT1*, thereby compromising ROS production [200]. In *N. benthamiana*, PsAvh113 interacts with the TFs NbMYB4 and NbERF114 (Figure 2), while also inducing global alterations in genes associated with stress responses and signaling pathways [201]. In parallel, another *P. sojae* effector, PsCRN108 (Figure 2), enhances the repressive activity of NbCAMTA2 (Figure 2), a negative regulator of immunity, thereby intensifying the suppression of genes such as NbHSP40 and reducing ROS production [202].

The manipulation of host TFs by filamentous pathogens may also occur through the modulation of hormonal pathways. In *Arabidopsis*, the oomycete *H. arabidopsidis* (Ha) secretes the effector HaRxL106 (Figure 2), which interacts with the TF BIM1 (Figure 2), a component of the brassinosteroid signaling pathway [203]. This nuclear interaction alters *BIM1* transcriptional activity, inducing BR-responsive genes and promoting growth-related changes, including shade-avoidance-like phenotypes. Consequently, the balance between growth and defense is disrupted, favoring host susceptibility [203].

In rust pathosystems, such as those involving *Puccinia triticina* (Pt), the effector Pt-1234 interacts with TaNAC069 (Figure 2), a positive regulator of resistance in wheat, by binding to its C subdomain and impairing its function [204]. As a result, ROS production is reduced, and defense responses are weakened, facilitating fungal colonization [204]. The relevance of this mechanism is further supported by the significant reduction in virulence observed when this effector is silenced through host-induced gene silencing (HIGS) [204].

Other fungi adopt functionally similar, although mechanistically distinct, strategies. In *B. cinerea* (Bc), the effector BcSCR1 represses the transcriptional activity of the TF PFB1 (Figure 2) in *Arabidopsis*, reducing the expression of JA-associated defense genes, such as *OPR3* and *WRKY33*, and thereby promoting infection [205]. Consistently, deletion of BcSCR1 reduces virulence, whereas its presence enhances host susceptibility [205]. In *Colletotrichum gloeosporioides*, the effector Vne1 directly interferes with the DNA-binding capacity of MdAHL17 (Figure 2) in apple (*Malus × domestica*), impairing the activation of resistance genes such as *MdCNL1*, which is associated with ROS production and programmed cell death. Consequently, immune responses are attenuated, and disease severity increases, underscoring the central role of this effector in pathogenicity [206].

The targeting of TFs is also a central strategy in bacterial pathogenicity, as illustrated by *R. solanacearum*, which employs distinct effectors to reprogram plant immunity through different mechanisms. In pepper, the effector RipAK compromises the function of the TF ERF098 (Figure 2) by inhibiting its homodimerization and DNA-binding capacity, thereby reducing the expression of defense-related genes (*PR1*, *DEF1*) and drought-tolerance genes (*OSM1*, *OSR1*), which increases susceptibility to diseases and water loss [207]. In *Arabidopsis*, the effector RipH1 of this pathogen acts in a complementary manner by interacting with the TF AtBBX31 (Figure 2), promoting its stabilization and relocation to the membrane, thereby compromising its nuclear function. Because AtBBX31 positively regulates defense genes such as *AtBBX29*, this interference prevents their transcriptional activation, thereby disrupting the *AtBBX31*-*AtBBX29* regulatory axis and suppressing immunity [208]. Collectively, these mechanisms demonstrate that *R. solanacearum* exploits both the direct inhibition of TF activity and the alteration of subcellular localization to promote infection [207,208].

Similarly, the bacterium *Candidatus Liberibacter asiaticus*, the causal agent of Huanglongbing, manipulates the citrus TF CsTCP15 through two complementary mechanisms [209]. The effector SECP8 interacts with CsTCP15 (Figure 2) in the nucleus, inhibiting its dimerization, a prerequisite for its activity, while simultaneously promoting its CsBRG3 E3 ligase-dependent ubiquitination and subsequent degradation by the 26S proteasome [209]. Because CsTCP15 activates SA pathway-related genes, such as *CsPR5* and *CsWRKY22*, its inactivation reduces their expression and weakens the immune response, thereby favoring bacterial colonization. This role is supported by functional evidence showing that *CsTCP15* overexpression enhances resistance, whereas its silencing increases susceptibility [209].

The manipulation of transcriptional machinery is not restricted to fungi and bacteria; it is also exploited by viruses and nematodes. In plant viruses, such as Tomato spotted wilt virus, infection is facilitated by the manipulation of TCP17, a key transcriptional regulator of auxin biosynthesis. TCP17 activates the expression of *YUCCA* (*YUC*) genes, which are essential for hormone production and defense responses. However, the viral effector NSs interferes with TCP17 dimerization (Figure 2), which is required for its transcriptional activity. This interference reduces *YUC* expression and auxin levels in the plant [210]. Therefore, hormonal signaling is suppressed, and immune responses are weakened, favoring infection. The conservation of this mechanism is supported by the fact that proteins from other viruses, including Barley stripe mosaic virus, Rice stripe virus, and Tomato bushy stunt virus, also interfere with TCP17 dimerization, suggesting a recurrent pattern of immune reprogramming through hormonal regulation [210].

In parallel, phytopathogenic nematodes also exploit host transcriptional regulators to promote parasitism. In *M. incognita* (Mi), the effector Mi2G02 targets GT-3a (Figure 2), a trihelix-family TF in *Arabidopsis* [211]. After being translocated into the nucleus, Mi2G02 interacts with GT-3a and inhibits its degradation via the 26S proteasome, thereby promoting its stabilization [211]. As a result, this TF, which functions as a transcriptional repressor, intensifies the repression of defense-associated genes, such as *TOZ* and *RAD23C*, leading to host transcriptional reprogramming. This modulation facilitates the formation of giant cells and feeding sites, which are essential for nematode nutrition, while also increasing plant susceptibility [211].

These findings demonstrate that the manipulation of TFs constitutes a central and conserved strategy among diverse pathogens, including fungi, bacteria, viruses, and nematodes. Supplementary Table S1 presents additional studies reporting newly identified effectors and the mechanisms by which they impair the proper function of TFs involved in plant defense responses.

## 6. Transcription Factors as Strategic Targets for Crop Immune Engineering: Opportunities, Trade-Offs, and Breeding Applications

**Figure 3:**
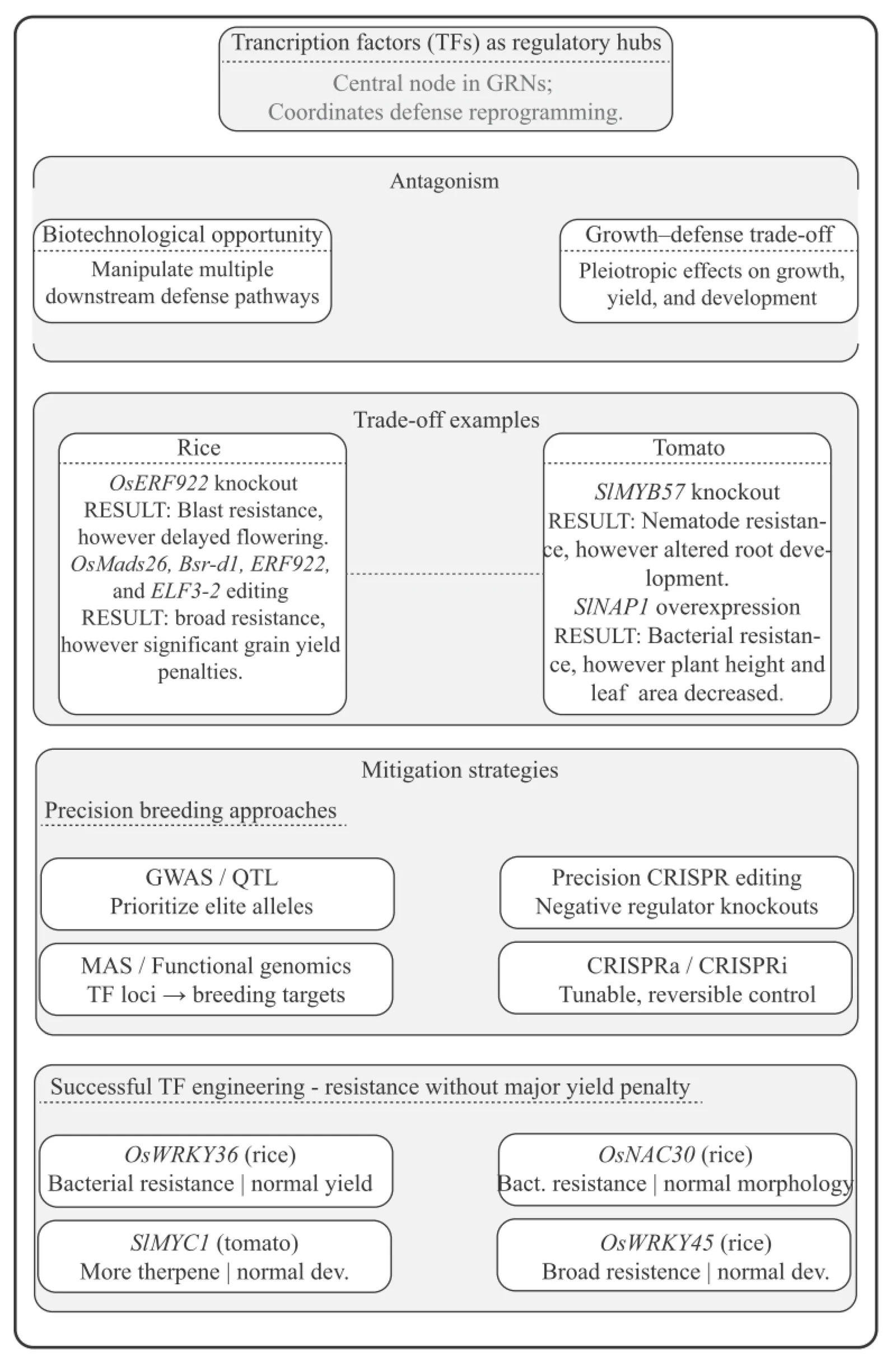
Transcription factor-centered framework for precision crop immune engineering. Transcription factors act as regulatory hubs in gene regulatory networks, enabling coordinated manipulation of multiple defense pathways, but also creating growth–defense trade-offs through pleiotropic effects on development and yield. Representative examples in rice and tomato (described in the main text) illustrate both adverse phenotypes and successful resistance engineering with limited agronomic penalties. Mitigation strategies include GWAS/QTL-based identification of favorable alleles, marker-assisted selection and functional genomics, precision CRISPR editing, and CRISPRa/CRISPRi-mediated control of endogenous TF expression. The resulting phenotypes may vary according to genetic background, gene target, and pathogen.

TFs have been recognized as strategic molecular targets for crop improvement because of their roles in coordinating plant immune responses [21]. In plant-pathogen interactions, TFs act not only as downstream regulators of defense gene expression but also as regulatory hubs capable of linking pathogen perception to broad transcriptional reprogramming (Figure 3). Accordingly, recent advances in genome-wide analyses and omics technologies have accelerated the identification and characterization of TF-associated alleles underlying agronomically important traits, thereby creating new opportunities for translational crop improvement [212,213,214,215].

Because TFs often occupy central positions within gene regulatory networks, as discussed in Section 7.1, their manipulation can simultaneously affect multiple downstream defense-associated pathways (Figure 3), as illustrated by the examples presented in Section 2 and its subsections. Although TF engineering is therefore a promising strategy for enhancing disease resistance, it may also generate trade-offs among immunity, plant growth, and productivity [216,217,218,219] (Figure 3). These trade-offs largely arise from the pleiotropic position of these regulators within plant gene regulatory networks. As shown in this review, defense-associated TFs frequently control not only immune genes but also pathways involved in primary metabolism, photosynthesis, flowering, and organ development, among others. Consequently, constitutive TF activation or complete loss of function may redirect resources toward defense-related metabolism, sustain ROS and hormone signaling, and restrict growth or reproductive investment even in the absence of pathogen pressure. The resulting phenotypes may include reduced biomass, altered plant architecture, delayed flowering, impaired photosynthetic performance, and lower grain or seed yield. Therefore, the agronomic cost of TF manipulation depends not only on whether immunity is enhanced but also on the magnitude, duration, tissue distribution, and developmental timing of the engineered transcriptional response.

Several studies across different crop species have demonstrated growth-defense trade-offs associated with TF engineering. In tomato (Figure 3), knockout of *SlMYB57* improved resistance to nematodes but negatively affected root development by disrupting flavonoid metabolism [220,221]. In the same species, SlNAP1 overexpression provides another representative example of the pleiotropic consequences associated with TF-based immune engineering [12]. Its overexpression enhanced resistance to *Pseudomonas syringae* pv. tomato DC3000 and *R. solanacearum*, but also caused growth and physiological alterations, including reduced plant height, leaf area, and photosynthetic rate [12]. In rice (Figure 3), multiplex editing of *OsMads26*, *OsBsr-d1*, *OsERF922*, and *OsELF3-2* generated broad-spectrum resistance against multiple pathogens, but also resulted in significant grain yield penalties [222]. Similarly, *OsERF922*-edited lines exhibiting enhanced blast resistance showed delayed flowering, altered panicle architecture, and reduced grain weight [223] (Figure 3). These examples highlight that enhanced immunity does not necessarily translate into agronomically favorable phenotypes, reinforcing the need for strategies that balance resistance with plant performance.

To reduce the risks associated with defense-related trade-offs, recent advances in GWAS, quantitative trait locus (QTL) mapping, and marker-assisted selection (MAS) have enabled more strategic identification of TF-associated alleles linked to disease resistance and agronomic stability (Figure 3). These approaches support prioritizing naturally occurring or elite TF variants capable of enhancing immunity while minimizing negative pleiotropic effects on plant growth and productivity [218]. By exploiting natural variation, these strategies favor alleles with moderate or context-dependent effects rather than constitutive TF activation or complete loss of function. This can preserve developmental and metabolic functions while maintaining sufficient immune responsiveness. Moreover, combining association data with multi-environment phenotyping allows breeders to exclude variants associated with reduced biomass, delayed flowering, or yield penalties. Thus, GWAS, QTL mapping, and MAS help decouple disease resistance from undesirable pleiotropic effects and support the introgression of more agronomically stable TF alleles into elite cultivars. In parallel, the integration of functional genomics into precision breeding pipelines has accelerated the discovery of TF-associated loci with direct translational relevance, allowing candidate regulators identified in omics studies to be more rapidly converted into targets for crop improvement [224].

Some studies illustrate the potential of these approaches for TF-guided crop improvement. In sorghum (*Sorghum bicolor*), GWAS analysis identified a WRKY associated with resistance to sugarcane aphid (*Melanaphis sacchari*), and subsequent functional analyses confirmed its role in insect resistance [225]. Similarly, QTL and association mapping studies on barley (*Hordeum vulgare*) identified HvWRKY6 as a candidate regulator involved in resistance-associated pathways against *Pyrenophora teres* f. teres [226]. In common bean (*Phaseolus vulgaris*), molecular markers associated with an *NAC*-linked resistance locus for Bean golden yellow mosaic virus have been integrated into marker-assisted breeding programs, facilitating the incorporation of virus resistance into elite breeding germplasm [227]. In soybean, joint linkage analysis and GWAS identified an elite natural haplotype of GmMYC3, encoding an MYC2 TF, which enhanced resistance to the common cutworm without significant reductions in seed yield or quality [228]. Functional and field analyses further demonstrated that GmMYC3 activates defense-related genes, including Kunitz-type trypsin inhibitors and GmWRKY56 [228].

In the precision breeding context, *OsERF922* (an AP2/ERF family member) represents an interesting example of translational engineering for disease resistance. CRISPR/Cas9-mediated knockout of *OsERF922*, a negative regulator of rice immunity, enhanced resistance to both rice blast, caused by *M. oryzae*, and bacterial blight, caused by *X. oryzae* pv. *oryzae*, through the activation of defense pathways associated with SA and JA. The edited lines maintained agronomic performance comparable to the wild type, with no significant differences in plant height, number of tillers, panicle length, or number of grains per panicle [229]. In maize, CRISPR/Cas9-induced loss of function of ZmWRKY125 substantially reduced *Fusarium verticillioides* ear rot severity and fumonisin contamination [230]. Transcriptomic and hormonal analyses indicated that ZmWRKY125 functions as a negative regulator of defense pathways involving JA, ABA, redox homeostasis, cell-wall modification, and secondary metabolism. Wild-type-like phenotypes were recorded in the edited lines [230]. These cases illustrate how precise disruption of negative immune regulators may enhance resistance while preserving key agronomic traits, although outcomes can vary depending on genetic background, target site, and editing strategy.

In tomato, *SlMYC1* has emerged as an important regulator of herbivore-associated defense metabolism (Figure 3). Functional analyses involving both CRISPR/Cas9-mediated knockout and *SlMYC1* overexpression demonstrated that this TF positively regulates the development of glandular trichomes and the biosynthesis of terpenes associated with constitutive defenses against herbivores. Mechanistically, *SlMYC1* participates in JA-associated regulatory pathways, controlling trichome formation and secondary metabolism linked to herbivore resistance. Importantly, plants overexpressing *SlMYC1* did not exhibit severe developmental abnormalities under the experimental conditions evaluated [231].

In rice, *OsbHLH34* has been identified as a promising positive regulator of defense responses against sheath blight caused by *R. solani*. Functional characterization through both overexpression and knockout approaches demonstrated that *OsbHLH34* positively modulates antifungal immunity by interacting with ERF-mediated signaling pathways and promoting the expression of downstream defense-associated genes. Importantly, the engineered lines did not display noticeable developmental abnormalities under the conditions evaluated [232]. This example reinforces the potential of bHLH-associated regulatory modules as targets for improving disease resistance without necessarily compromising plant development.

*SlMYBS2* has emerged as a promising positive regulator of defense responses against late blight caused by *P. infestans* in tomato [233]. Functional analyses demonstrated that *SlMYBS2* positively modulates immunity-associated pathways involved in pathogen resistance, including the regulation of defense-related genes and ROS-mediated responses. Deletion of the *SlMYBS2* gene increased susceptibility to *P. infestans*, reinforcing its role as a positive regulator of tomato immunity. Importantly, the genetically modified plants did not show detectable reductions in growth or productivity under the experimental conditions evaluated [233].

Despite the extensive research on NAC TFs in plant stress responses, clear translational examples demonstrating enhanced resistance to biotic stresses without compromising agronomic performance remain comparatively limited. In this context, *OsNAC30* represents one of the most promising examples of NAC-based translational engineering in rice (Figure 3). CRISPR/Cas9-mediated disruption of *OsNAC30*, identified as a negative regulator of rice immunity, enhanced resistance against *X. oryzae* pv. *oryzae* while maintaining key agronomic traits, including plant height, panicle number, grain weight, and overall vegetative development, at levels comparable to wild-type plants [14]. Functional analyses further demonstrated that *OsNAC30* regulates immune-associated pathways linked to ROS accumulation and defense-related gene expression [14].

One of the most compelling examples of successful WRKY-based translational engineering has recently been reported for *OsWRKY36* in rice [224]. Multiplex CRISPR/Cas-mediated editing of *OsWRKY36*, identified as a negative regulator of rice immunity, conferred broad-spectrum resistance against rice blast caused by *M. oryzae* and bacterial blight caused by *X. oryzae* pv. *oryzae* [224] (Figure 3). Importantly, the edited elite rice lines maintained satisfactory agronomic performance, including normal vegetative growth, panicle development, and grain yield under the evaluated conditions, indicating minimal fitness costs associated with the engineered resistance [224] (Figure 3). Functional analyses further revealed that *OsWRKY36* participates in the transcriptional regulation of immune-associated pathways, highlighting the central role of WRKY-mediated regulatory networks in coordinating broad-spectrum defense responses [224].

Additional evidence supporting the translational relevance of WRKY-associated regulators has also been reported for *OsWRKY45* in rice [220] (Figure 3). This TF functions as a central positive regulator of SA-mediated immune signaling, coordinating the activation of downstream defense-associated genes involved in resistance to important fungal and bacterial pathogens, including *M. oryzae* and *X. oryzae* pv. *oryzae*. Importantly, controlled activation of *OsWRKY45*-associated pathways enhanced broad-spectrum disease resistance while minimizing detrimental effects on plant growth and productivity, demonstrating that precise modulation of immune-associated TF pathways can partially mitigate the classical growth–defense trade-off in crop plants [220] (Figure 3).

Similarly, CRISPR/Cas9-mediated knockout of *CsWRKY22* in sweet orange (*Citrus × sinensis*) significantly reduced susceptibility to citrus canker caused by *Xanthomonas citri subsp. citri* without detectable developmental abnormalities following grafting and vegetative growth evaluations [234]. In wheat, functional characterization of *TaWRKY19* demonstrated that this TF acts as a negative regulator of ROS-mediated immune responses, and its disruption enhanced resistance to stripe rust caused by *Puccinia striiformis* f. sp. tritici [235]. Importantly, the edited wheat lines did not exhibit severe developmental abnormalities under the evaluated conditions [235]. These studies further support the idea that targeting negative regulators of immunity can be an effective strategy to improve disease resistance, provided that the edited regulatory nodes do not strongly compromise development or yield-related traits.

Finally, to mitigate the fitness costs of constitutive defense activation via overexpression, emerging precision breeding strategies increasingly rely on programmable and finely tuned regulatory systems. These include inducible and tissue-specific promoters, multiplex genome editing, and synthetic regulatory elements designed to control immune responses in a spatially and temporally restricted manner [236]. Particularly promising are CRISPRa/CRISPRi-based platforms, which use catalytically inactive Cas proteins to activate or repress endogenous genes without introducing permanent genetic modifications [237,238,239]. By enabling quantitative and reversible control of transcriptional networks, these technologies allow precise adjustment of defense responses while minimizing pleiotropic effects associated with constitutive immune activation. Together, advances in plant synthetic biology, functional genomics, and TF-based precision breeding are paving the way for next-generation disease-resistance strategies capable of reducing growth-defense trade-offs while preserving agronomic performance.

## 7. Future Perspectives and Remaining Challenges in TF Studies

The biotechnological and translational actions described in the preceding section, wherein TF-based strategies have been leveraged to improve resistance to pathogens, do not operate in isolation from the methodological and conceptual frontiers that are actively shaping the field. As multi-omics platforms mature and the regulatory complexity of plant genomes becomes progressively better resolved, the study of TFs is entering a phase defined not merely by the characterization of individual regulators but by the systemic mapping of the networks within which they operate. This transition demands new theoretical frameworks, integrative computational architectures, and a willingness to engage with genomic regions whose regulatory significance was long underestimated. Additionally, such a transition gives rise to new challenges.

### 7.1. Systems Biology and Integrative Omics

The multi-omics era has generated substantial volumes of genomic, epigenomic, transcriptomic, proteomic, and single-cell omics data for diverse crop species, creating both an opportunity and an interpretive challenge for TF research. The shift toward gene regulatory network (GRN) inference represents perhaps the most consequential methodological development in this context. GRNs represent the regulatory links between TFs and their target genes, describing physical and regulatory interactions [240]. In plants, they are essential for understanding transcriptional programs that control important agricultural traits such as yield or plant-pathogen interactions. Rather than treating TFs as isolated molecular switches, system-level approaches position them as nodes within complex, hierarchical networks whose topology encodes molecular and environmental information.

Searches of scientific literature databases reveal some examples of GRNs associated with plant-microorganism interactions, including both pathogenic and non-pathogenic associations. Timmermann et al. [241] reconstructed a GRN to model induced systemic resistance in *A. thaliana* triggered by the beneficial bacterium *Paraburkholderia phytofirmans* PsJN during subsequent infection with *P. syringae* DC3000. Five of the eight genes included in the network encode TFs: *WRKY70*, *WRKY54*, *WRKY33*, *MYC2*, and *ERF1*. These elements form a regulatory backbone that coordinates the balance between SA-mediated and JA/ET-mediated immune responses. The network analysis suggested that *WRKY70*, *MYC2*, and *ERF1* were particularly important for maintaining the dynamic behavior of the inferred defense network, since the combined removal of these nodes severely disrupted the Boolean trajectory representing the biological response. The study highlighted the central role of TFs in shaping the regulatory architecture of induced systemic resistance and provided a computational framework for generating hypotheses about transcriptional control in plant defense [241].

In the GRN reconstructed by Sánchez et al. [242] for the *A. thaliana*-*Trichoderma atroviride* mutualistic interaction, TFs emerged as central regulators of the coordinated transcriptional reprogramming occurring in both partners. In *A. thaliana*, the interaction-specific GRN was enriched in TF-centered modules, particularly involving members of the ERF, WRKY, NAC, DOF, MYB, GATA, bZIP, bHLH, and G2-like families. These regulators were mainly associated with two major biological processes: hypoxia-related responses and root developmental remodeling. ERF factors, including *ERF73*/*HRE1*, were linked to low-oxygen acclimation, ABA/ROS signaling, and stress adaptation, whereas WRKY factors formed a defense-associated module integrating immune- and hormone-related responses. NAC and DOF regulators, such as *NTM1*, *DOF1*/*DOF1.7*, and *OBP3*, connected stress signaling with nitrogen metabolism, developmental regulation, and root architecture plasticity [242].

In the study by Wang and Zhang [243], TFs were identified as relevant components of the dynamic network associated with the MeJA-induced growth-to-defense transition in *A. thaliana*. Rather than reconstructing a classical causal TF-target GRN, the authors used a dynamic network biomarker approach to identify genes associated with the critical transition point, followed by a core network connecting these biomarkers to differentially expressed genes. Several TFs were detected among the dynamic network biomarker genes, including *bZIP63*, *HAT22*, *ERF043*, *CRF6*, *BPC1*, *MYB3R-5*, *AT5G26865*, and *AT3G58630*. *bZIP63* was associated with stress and energy-related signaling, while HAT22 was linked to stress responses and growth restriction. ERF043 was associated with JA-related defense responses, whereas CRF6 was linked to cytokinin, stress, and developmental signaling. *MYB3R-5* was linked to growth inhibition through repression of mitotic genes. Additionally, *WRKY33* emerged as a hub in the core network, reinforcing its role in plant defense. Overall, the study suggested that MeJA-induced defense transition involves coordinated transcriptional regulation, linking hormonal signaling, metabolic reprogramming, growth suppression, and immune activation [243].

The application of these methods, however, remains limited in plant species [219], reflecting a pronounced asymmetry between the methodological sophistication developed in biomedical research and the data infrastructure available to crop science. A further limitation is that most current GRN inference methods do not specify whether a given TF activates or represses its target genes, a distinction that may hinder a full mechanistic understanding of TF function and impose limitations on downstream applications. This limitation, however, was recently resolved in the work of Ren et al. [244], who developed the *ScSAGRN* framework, which can identify key activating and repressive TFs.

### 7.2. The Integrative Role of “Genomic Dark Matter” and Transcription Factors

A second scientific frontier concerns the regulatory layers that operate upstream of, or in concert with, classical TF activity. The non-coding fraction of eukaryotic genomes has emerged as a structurally and functionally integral component of gene regulation. Up to 90% of eukaryotic genomes are estimated to be transcribed, yet only approximately 2% of transcribed RNAs encode proteins [245]. The non-coding genome, also referred to as the “dark matter” [246], includes, among others, non-coding RNAs (ncRNAs), whose biological functions have become increasingly significant. Some studies addressing the important interplay between TFs and ncRNAs in the context of plant-pathogen interactions are discussed in Section 3.2.

However, despite the conceptual significance of these interactions, the role of ncRNAs in plants remains largely unexplored, with functional characterization progressing more slowly than their identification. The vast majority of plant lncRNAs detected by omics surveys lack experimental validation [247], and the regulatory networks in which they participate remain poorly resolved. Integrating ncRNA biology into TF research, as a layer of regulation rather than a background signal, constitutes one of the most significant priorities in plant molecular biology.

### 7.3. Transcription Factors as Tools for Pathogen-Resistant Crops

The molecular and physiological mechanisms by which plants respond to pathogens have become considerably better understood through advances in integrative analyses, which underscore the critical role of transcriptional regulation in managing stress responses. The capacity of individual TFs to simultaneously regulate multiple stress pathways (e.g., *MdMYB88* and *MdMYB124* [248] and *GhMYB36* [249]) has rendered them attractive targets for engineering.

Transgenic and, in particular, genome-editing approaches have substantially refined our ability to manipulate TF activity with precision, as reported in Section 6. Despite these advances, the field still faces important conceptual and methodological limitations. The predominance of studies conducted in model organisms, particularly *A. thaliana* (as highlighted in Section 2), and a small number of well-resourced crops, such as tomato and rice (as seen in Section 6), continues to constrain the generalizability of mechanistic conclusions. TF families are taxonomically conserved in their domain architecture but often functionally divergent across species, and regulatory networks reconstructed in model systems may not faithfully represent the regulatory landscape of crops with complex genome architectures and heterogeneous chromatin organization. A recent example of this issue is provided by Galli et al. [250]. In that study, the authors mapped the binding profiles of 200 TFs from 30 families across two maize inbred lines and identified widespread genotype-specific differences, largely driven by structural variation, that correlated with changes in gene expression. These findings demonstrate that even within a single crop species, regulatory networks inferred from a reference genotype may not faithfully represent the regulatory architecture of other genotypes.

### 7.4. Gaps in Current Knowledge and Priorities for Research

As discussed in Section 6, the pleiotropic nature of TFs remains a relevant challenge for translational applications [251]. In addition to regulating stress-responsive networks, these proteins frequently govern developmental processes, and their ectopic overexpression or constitutive editing may produce unintended phenotypic consequences, including dwarfing, late flowering, and lower yields [252,253]. To predict such potentially undesirable phenotypic effects, deep learning models may become an important alternative. However, although these models can capture complex patterns in GRN inference, they often require large training datasets and substantial computational resources. They may also be less interpretable than traditional statistical models [254]. This limitation complicates both mechanistic interpretation and regulatory scrutiny. The interpretability deficit of black-box deep learning architectures is not merely a technical inconvenience but a knowledge-production problem: if researchers cannot identify which features of the regulatory landscape drive model predictions, the biological insights that can be derived from those predictions remain fundamentally constrained.

The future of TF research will be shaped by the expanding power of integrative omics and computational inference. It will also depend on recognizing that regulatory networks extend beyond protein-coding boundaries and translating molecular knowledge into crop varieties capable of withstanding future climatic and biotic pressures. The convergence of these dimensions (mechanistic, computational, and agronomic) demands not only technical innovation but an intellectual framework capable of managing the complexity they collectively present. It is within this framework that the broader conclusions of the present review must be situated as the field moves from cataloging what TFs do to understanding, predicting, and engineering what they can become.

## 8. A TF-Centered Framework for Plant Immunity

**Figure 4:**
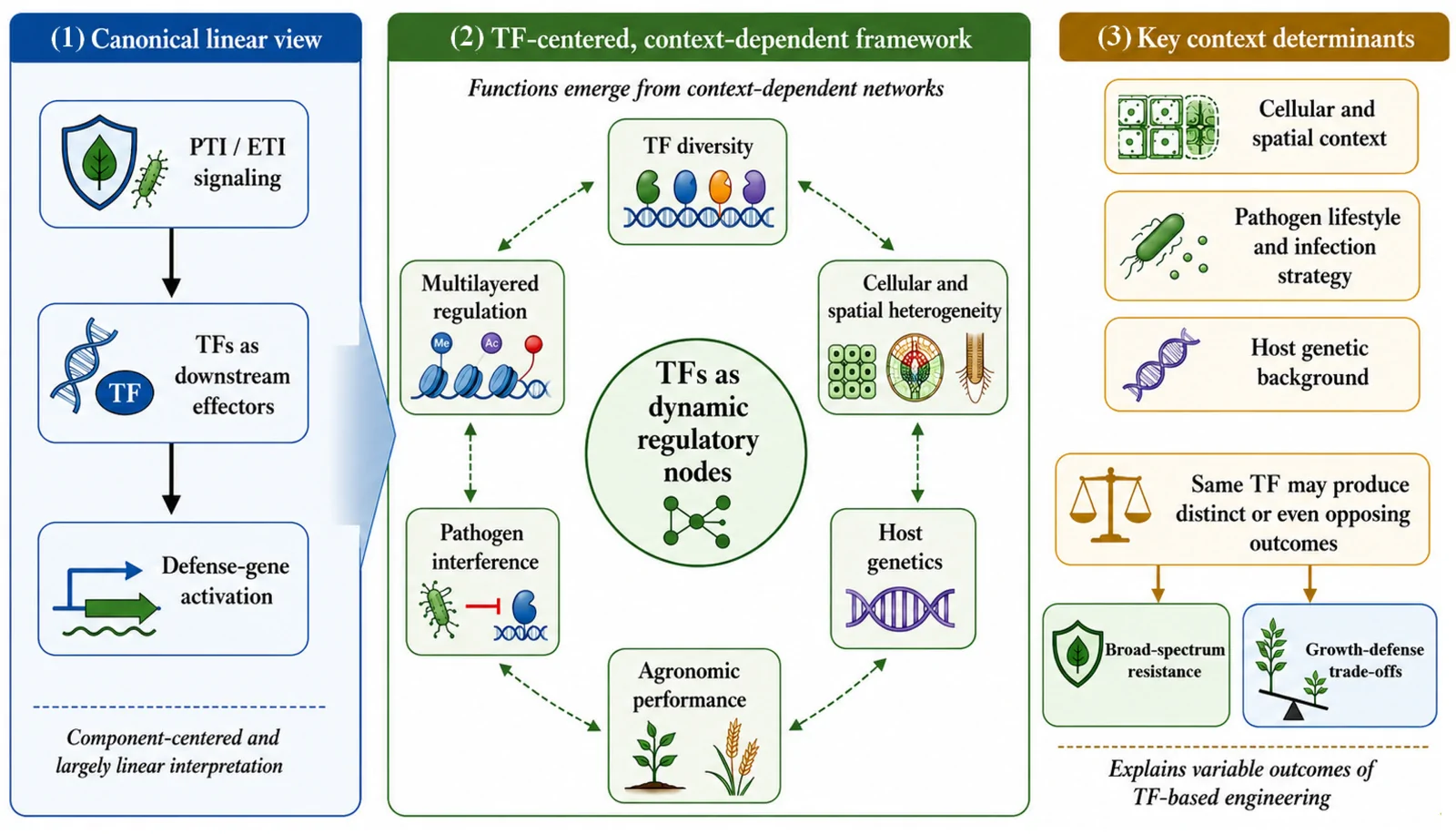
Presentation of a transcription factor-centered, context-dependent framework for plant immunity. The scheme expands the canonical linear view (1), in which transcription factors (TFs) are primarily positioned as downstream effectors of PTI and ETI signaling, by incorporating (2) TF diversity, multilayered regulation, cellular and spatial heterogeneity, pathogen interference, and host genetics into a dynamic regulatory network. Cellular context, pathogen lifestyle, and host genetic background influence whether a given TF promotes broad-spectrum resistance or generates undesirable growth–defense trade-offs, highlighting the relevance of context-dependent TF regulation for precision crop engineering (3). Abbreviations: ETI, effector-triggered immunity; PTI, pattern-triggered immunity.

The synthesis of the evidence examined in this review enables us to outline a TF-centered framework for plant immunity (Figure 4). Building on canonical linear models in which TFs are portrayed merely as downstream effectors linking PTI or ETI signaling to defense-gene activation, this framework extends the prevailing view by positioning TFs as dynamic regulatory nodes whose functions emerge from context-dependent networks (Figure 4). Accordingly, the biological output of a given TF cannot be inferred solely from its family identity or its classification as a positive or negative immune regulator. Rather, it results from the combined influence of the cellular and spatial context in which the TF operates, the lifestyle and infection strategy of the pathogen, and the genetic background of the host (Figure 4). Thus, the same TF may produce distinct or even opposing outcomes depending on the cell type, pathogen, and signaling environment. This context dependence, together with variation in host genetic background, provides a biological explanation for why TF-based engineering may confer broad-spectrum resistance in some cases while generating undesirable growth–defense trade-offs in others (Figure 4).

Although the proposed framework is not intended as a single mechanistic or quantitative model, it extends previous representations by linking TF diversity, multilayered regulation, cellular and spatial heterogeneity, pathogen interference, host genetics, and agronomic performance (Figure 4). It therefore shifts the interpretation of TF-mediated immunity from a primarily linear and component-centered view toward a dynamic, network-based, and context-dependent perspective with direct implications for precision crop engineering (Figure 4).

## 9. Conclusions

The evidence synthesized in this review supports a view of plant defense in which TFs are not merely downstream executors of immune signaling but dynamic regulatory actors that determine how pathogen perception is translated into developmentally compatible, tissue-specific, and metabolically coordinated defense programs. Across established families such as WRKY, MYB, AP2/ERF, bHLH/MYC, and NAC, as well as emerging groups including NF-Y, Trihelix, PLATZ, TCP, and GRAS, a common principle becomes evident: immune competence depends on the precise modulation of transcriptional networks rather than on the isolated activation of single resistance genes.

The main contribution of this review is to present recent evidence on TF multilayered regulation, cell-resolved and spatial transcriptomics, pathogen effector activity, and translational crop engineering and its challenges within a conceptual framework. This synthesis shifts the interpretation of TF-mediated immunity from a primarily linear and component-centered view toward a dynamic, network-based, and context-dependent perspective with direct implications for precision crop engineering.

Despite several advances, important limitations remain. Current knowledge is still disproportionately derived from Arabidopsis and a restricted set of major crops, and many inferred TF-target relationships lack functional validation in relevant genotypes, tissues, developmental stages, and field-like conditions. Moreover, the pleiotropic nature of TFs complicates direct translational use, since enhanced immunity may be accompanied by penalties in growth, reproduction, or yield. Computational inference of gene regulatory networks, although increasingly powerful, also requires greater interpretability, causal validation, and integration with non-coding regulatory layers.

Future progress will therefore depend on combining cell- and spatially resolved multi-omics, comparative genomics, natural variation, perturbation-based functional assays, and precision breeding. Particularly promising are strategies that modulate TF activity quantitatively, conditionally, or tissue-specifically, including inducible promoters, CRISPRa/CRISPRi platforms, synthetic regulatory elements, and allele-informed breeding. In summary, TF-centered research is moving from cataloging defense regulators toward predicting and engineering immune network behavior. This transition will be essential for developing crops with durable pathogen resistance, reduced reliance on chemical control, and improved resilience under increasingly complex agricultural environments.

## Figures and Tables

**Table 1:** Summary of the major transcription factor (TF) families associated with plant defense identified in the NLP-based corpus analysis, with their principal biological functions, representative target genes, and associated signaling pathways.

TF Family	Principal Biological Functions	Representative Target Genes	Associated Signaling Pathways
WRKY	Regulation of PTI and ETI; activation or repression of defense genes; coordination of hormonal, metabolic, stomatal, and structural defenses	*ICS1*, *PBS3*, *NRG1*, *PR1/NtPR1-L1*, *NPR1*, *DEF1*, *WRKY30IIc*, *LOX5*, *PAL*, *C4H*, and lignin-biosynthesis genes	MAPK cascades; SA and JA signaling; PAMP/PTI and NLR/ETI signaling; phenylpropanoid and phytoalexin biosynthesis; callose deposition; growth–immunity crosstalk
MYB [R2R3-class (predominant)]	Activation of lignin biosynthesis and cell-wall reinforcement; regulation of hormone-mediated immunity; coordination of growth–defense trade-offs; activation of anthocyanin and defensin responses	*4CL1/2*, *CAD3*, *LACs*, *SPL9*, *DFR1*, *EDS1*, *PDF1.2*, and lignin-biosynthesis genes	Lignin/phenylpropanoid pathway; SA, JA, and ET signaling; MAPK3/6 signaling; gibberellin–DELLA pathway; anthocyanin biosynthesis; miRNA-mediated regulation
ERF/AP2 AP2	Regulation of defense against necrotrophs, biotrophs, herbivores, and nematodes; antimicrobial metabolite production; stomatal defense; context-dependent activation or repression of immunity	*ACT*, *ERF1b*, *ERF113*, *PR1*, *WRKY67*, *ERF083*, *PR2*, *PR5*, *PR10-09g*	ET and JA signaling; SA–JA crosstalk; MAPK signaling; EIN3/EIL–ERF pathway; oxygen sensing and N-end rule pathway; phytoalexin biosynthesis
bHLH/MYC	Regulation of herbivory, wound, and pathogen defenses; coordination of specialized metabolism and growth–defense trade-offs; antiviral and tissue-specific immune regulation	*PUB22* and genes involved in tryptophan, indole glucosinolate, and anthocyanin biosynthesis	COI1–JAZ–MYC jasmonate signaling; MED25–MYC–MYB complexes; ubiquitin–26S proteasome pathway; tryptophan and glucosinolate metabolism
NAC	Activation of pathogen and herbivore defenses; regulation of phytohormone and defensive volatile biosynthesis; membrane-dependent immune activation; prevention of excessive immune responses	*ICS1*, *LOX2*, *BAHD acyltransferase*, *ALD1*, *FMO1*	SA, JA, and NHP signaling; BAK1-dependent immunity; ER–Golgi–nucleus translocation; defensive volatile biosynthesis

Legend: Principal functions and target genes are representative, not exhaustive, and are grounded exclusively in the literature cited in the present review. Abbreviations: BAK1, BRASSINOSTEROID INSENSITIVE 1-Associated Receptor Kinase 1; COI1, Coronatine Insensitive 1; DELLA, Aspartic acid–Glutamic acid–Leucine–Leucine–Alanine; EIL, Ethylene Insensitive 3-Like; EIN3, Ethylene Insensitive 3; ER, Endoplasmic Reticulum; ERF, Ethylene Response Factor; ET, Ethylene; ETI, Effector-Triggered Immunity; JA, Jasmonic Acid; JAZ, Jasmonate ZIM-Domain; MAPK, Mitogen-Activated Protein Kinase; MED25, Mediator Complex Subunit 25; miRNA, MicroRNA; MYB, Myeloblastosis; MYC, Myelocytomatosis; NHP, N-Hydroxypipecolic Acid; NLR, Nucleotide-Binding Leucine-Rich Repeat Receptor; PAMP, Pathogen-Associated Molecular Pattern; PTI, Pattern-Triggered Immunity; SA, Salicylic Acid; and ZIM, Zinc-Finger Inflorescence Meristem.

**Table 2:** Recent high-resolution studies (single-cell sequencing and/or spatiotemporal analyses) investigating plant immunity and cellular dynamics associated with modulation of transcription factor expression. Legend: TFs (transcription factors).

Reference	Technology	Plant–Pathogen System	Key TFs	Main Findings
Tan et al. [196]	scRNA-Seq	*A. thaliana/Colletotrichum higginsianum*	*MYB122*, *MYB51*, *MYB34*, *WRKY75*	*MYB122* regulates indole glucosinolate biosynthesis in epidermal cells; mutant lines exhibit hypersusceptibility to infection.
Cao et al. [197]	scRNA-Seq	*Zea mays*–*Fusarium verticillioides*	*ZmWOX5b*	*ZmWOX5b*, a homeobox TF, coordinates the auxin gradient by regulating ZmYUC6 to protect the root apical meristem (RAM) against fungal colonization; overexpression enhances resistance, whereas silencing increases susceptibility.
Sun et al. [198]	Spatial transcriptomics (Visium)	*Asparagus officinalis*–*Phomopsis asparagi*	*AoWRKY53*, *AoWRKY33-1*, *AoWRKY70-2*, *AoNAC68-0*, *AoNAC68-1*	WRKY TFs emerged as major regulators of cell-type-specific immune reprogramming during *P. asparagi* infection, coordinating SA/JA signaling, reactive oxygen species (ROS) homeostasis, lignification, detoxification, and systemic acquired resistance (SAR)-related responses across multiple stem tissues; in contrast, *AoNAC68* was predominantly associated with later developmental responses in distal tissues.
Li et al. [199]	Spatial transcriptomics (Stereo-seq)	*Solanum tuberosum*–*P. infestans*	*StWRKY75*, *StWRKY70*, *StWRKY41*, *StATAF1*, *StNAC042*	*StWRKY75* was identified as a central TF regulating SA/JA signaling, stomatal closure, and SAR in SPCs; overexpression enhanced resistance, whereas silencing increased susceptibility.

## Data Availability

No new data were created or analyzed in this study. Data sharing is not applicable to this article.

## Supplementary Materials

The following supporting information can be downloaded at: https://www.mdpi.com/article/10.3390/ijms27167315/s1.
