# Supplementary material for: The Regulatory Army of Plant Defense: Transcription Factors in the War for Plant Immunity
Source: Int J Mol Sci. 2026 Aug 16;27(16):7315. doi: 10.3390/ijms27167315 (PMC13513287; doi:10.3390/ijms27167315)
Supplement: Supplementary file 1 [file ijms-27-07315-s001.zip › Supplementary Material S1.pdf]

Corpus Description and Data Retrieval

The textual corpus used in the present study comprised 1,647 abstracts of peer-reviewed scientific articles retrieved from the PubMed database (https://pubmed.ncbi.nlm.nih.gov/). Records were collected through a structured Boolean search strategy using the following keywords: "transcription factors AND plant defense NOT review". The search encompassed publications from the last five years and was restricted to articles written in English. The full dataset was exported in plain-text (.txt) format from the PubMed interface, with each record containing bibliographic metadata (authors, journal, year, DOI, PMID) and the corresponding abstract. The resulting file served as the sole input for the subsequent computational analysis.

Text Preprocessing

Prior to term extraction, the raw corpus underwent a structured preprocessing pipeline implemented in Python (version 3.0) using the *re* (regular expression) standard library module. The full-text file was first parsed to segment individual records using record boundary detection based on the numbered citation format employed by PubMed exports (i.e., each record initiated with a sequential integer followed by a period and the journal title). This parsing step yielded 1,712 delimited text blocks, including 1,647 target abstracts along with associated metadata fields (author affiliations, conflict-of-interest statements, and bibliographic identifiers).

No case folding to lowercase was applied universally, as the nomenclature of transcription factor families in plant biology is highly case-sensitive (e.g., *WRKY*, *bZIP*, *bHLH*). Instead, pattern matching was performed using case-insensitive flags selectively, or case-sensitive matching where the target acronym had potential for false-positive overlap with common English words or chemical compound names. Metadata fields (e.g., author names, institutional affiliations, DOI strings, PMID identifiers, and conflict-of-interest statements) were not explicitly stripped prior to matching; however, given the specificity of the regular expression patterns employed, spurious matches from non-abstract sections were considered negligible and are expected to have a minimal effect on the overall frequency estimates.

Construction of a Controlled Vocabulary for Transcription Factor Families

A domain-specific controlled vocabulary was manually curated to capture the nomenclature of transcription factor (TF) families as employed in the plant science literature. The vocabulary was organized into 32 TF family entries, each associated with a set of regular expression patterns designed to detect canonical acronyms, common name variants, alternative spellings, and synonymous designations reported in the scientific literature. Representative examples of the nomenclature variants incorporated include:

- *ERF/AP2*, encompassing *APETALA2* (AP2), *Ethylene Response Factor* (ERF), *Dehydration-Responsive Element-Binding* (DREB), and the composite notation AP2/ERF;
- *MYC/bHLH*, including *basic helix-loop-helix*, MYC2, and bHLH;
- *NAC*, derived from the founding members *NAM*, *ATAF1/2*, and *CUC2*;
- *bZIP*, including the expanded forms *basic leucine zipper* and *basic-leucine zipper*;
- *EIL/EIN3*, covering *EIN3*, *EIL1*, and *Ethylene Insensitive 3*. Families with a high risk of false-positive matching.

Particularly those whose acronyms coincide with common abbreviations in adjacent biomedical fields (e.g., ARF as auxin response factor versus other uses; TCP; GATA) – were assigned narrower, context-anchored patterns that required co-occurrence with contextual tokens such as *"transcription factor"*, *"domain"*, *"family"*, or a flanking numeral, thereby reducing the incidence of spurious matches.

Term Identification Strategy

Term identification was performed through regular expression (regex) pattern matching applied independently to each text block (record) in the corpus. Each TF family entry in the controlled vocabulary was assigned a list of one or more compiled regex patterns. For every record, the search was conducted sequentially across all patterns associated with a given family. Word-boundary anchors (*\b*) were enforced on both sides of each target token to prevent partial-word matches (e.g., to avoid matching *WRKY* within a hypothetical compound string). Where appropriate, patterns were extended with look-ahead or contextual constraints to disambiguate acronyms that are polysemous across disciplines. The matching algorithm was implemented using Python's *re.search()* function, with flags adjusted on a per-pattern basis (*re.IGNORECASE* for case-insensitive families; no flags for case-sensitive targets). No natural language parsing, part-of-speech tagging, or dependency parsing was employed; the identification was based exclusively on string-level pattern matching.

Counting Criterion and Unit of Analysis

The unit of analysis adopted in this study was the individual abstract, equated to a single published article. For each TF family, the frequency metric reported is the number of distinct abstracts in which at least one match was detected, regardless of how many times the corresponding term or its variants appeared within that abstract. This binary per-abstract scoring approach, sometimes referred to as document frequency in the information retrieval literature, was deliberately chosen over raw term frequency (i.e., total occurrences across the corpus) to provide an estimate of the breadth of scientific interest in each TF family, as measured by the number of independent research works that mention it.

Consequently, multiple occurrences of the same TF family term within a single abstract were counted as one, and the same abstract could contribute to the count of multiple TF families if more than one family were mentioned therein.

Ranking and Classification of Results

Following the frequency count procedure, all 32 identified TF families were ranked in descending order of document frequency. The ten families exhibiting the highest document frequency values were classified as the *most frequently cited* TF families in the corpus. Conversely, TF families with the lowest non-zero document frequencies were identified as the *least frequently cited*; specifically, the ten families with the smallest document frequency scores were selected for this category. In cases of ties in frequency at the boundary positions of either ranking group, all tied families were retained. TF families for which no matching record was identified in the corpus were excluded from the final ranked list, as their absence precluded any inference regarding their representation in the literature associated with the defined search strategy.

Limitations of the NLP-analysis performed

Despite its utility for large-scale corpus screening, the NLP approach employed here carries inherent limitations that should be acknowledged. First, term identification relied exclusively on regular expression pattern matching applied to abstract-level text, without recourse to deeper linguistic processing such as part-of-speech tagging, named entity recognition (NER), or dependency parsing.

Second, although the controlled vocabulary was designed to capture common acronyms, full names, and established synonyms for each family, variant nomenclatures not represented in the dictionary may have produced false negatives; conversely, polysemous acronyms (e.g., ARF, TCP, GATA) carry a residual risk of false-positive matches from unrelated biomedical contexts.

Third, because the analysis was restricted to abstracts rather than full-text articles, TF families mentioned exclusively in the introduction, results, or discussion sections of the original papers are not captured. Finally, the document frequency metric adopted here should not be interpreted as a direct proxy for biological relevance or functional importance. TF families that currently remain underrepresented in the literature may nonetheless emerge as critical regulatory nodes in plant immunity as future research further elucidates their functions. These limitations notwithstanding, the analysis provides a reproducible, quantitative overview of the relative representation of TF families in the plant defense literature indexed in PubMed/NCBI.
