# Supplementary material for: The Regulatory Army of Plant Defense: Transcription Factors in the War for Plant Immunity
Source: Int J Mol Sci. 2026 Aug 16;27(16):7315. doi: 10.3390/ijms27167315 (PMC13513287; doi:10.3390/ijms27167315)
Supplement: Supplementary file 1 [file ijms-27-07315-s001.zip › Supplementary Table S1.pdf]

Supplementary Table S1. Studies addressing plant-pathogen interactions, with emphasis on how pathogen-secreted molecules target plant transcription factors (TFs) and reshape host gene expression programs, thereby compromising defense responses and favoring infection establishment.

Pathogen	Effector / Molecule	Target TFs	Plant	Mechanism of Interference	Reference
Tobacco mosaic virus	Replicase	*ATAF2* (NAC)	Arabidopsis	Promotes TF degradation	[1]
Phytophthora infestans	Pi03192	*NTP1/2* (NAC)	Potato / *N. benthamiana*	Blocks nuclear TF relocalization	[2]
Xanthomonas euvesicatoria	XopD	*SlERF4*	Tomato	Destabilizes TF (desumoylation)	[3]
Pseudomonas syringae	HopD1	*NTL9* (NAC)	Arabidopsis	Suppresses TF-mediated immunity	[4]
Ralstonia / Pseudomonas	PopP2 / AvrRps4	*WRKY*	Arabidopsis	Acetylation / DNA-binding interference	[5]
Phakopsora pachyrhizi	PpEC23	*GmSPL12l*	Soybean	Suppresses TF immune function	[6]
Pseudomonas syringae	HopBB1	*TCP14*	Arabidopsis	Promotes proteasomal TF degradation	[7]
Phytophthora infestans	AVR2	*StCHL1* (bHLH)	Potato	Activates susceptibility TF	[8]
Verticillium dahliae	VdSCP41	*CBP60g / SARD1*	Arabidopsis	Inhibits TF transcriptional activity	[9]
Bremia lactucae	RxLR effectors	*LsNAC069*	Lettuce	Blocks nuclear TF relocalization	[10]
Hyaloperonospora arabidopsidis	HaRxLL470	*HY5* (bZIP)	Arabidopsis	Reduces TF DNA-binding activity	[11]
Ralstonia solanacearum	RipAB	*TGA*	Arabidopsis / Tomato	Blocks RNA Pol II recruitment	[12]
Xanthomonas campestris	TAL effectors	*ERF121*	*Brassica oleracea*	Activates TF and enhances susceptibility	[13]
Phytoplasma	SAP05	*SPL / GATA*	Arabidopsis	Induces TF degradation	[14]
Phytoplasma mali	SAP11-like	*TCP*	Arabidopsis	Destabilizes TCPs	[15]
Colletotrichum gloeosporioides	CgNLP1	*HbMYB8-like*	Rubber tree	Disrupts TF nuclear localization	[16]
Puccinia striiformis	Pst21674	*TaASR3*	Wheat	Blocks TF polymerization	[17]
Ascochyta rabiei	ArPEC25	*CaβLIM1a*	Chickpea	Inhibits TF DNA binding	[18]
Phytoplasma solani	SAP11-like	*AtTCP2/4*	Arabidopsis	Destabilizes TCPs	[19]
Magnaporthe oryzae	AvrPik-D	*WG7*	Rice	Enhances TF activity (high susceptibility)	[20]
Verticillium dahliae	Vd6317	*AtNAC53*	Arabidopsis	Inhibits TF transcriptional activation	[21]

Arabidopsis (*Arabidopsis thaliana*), potato (*Solanum tuberosum*), bentham's tobacco (*Nicotiana benthamiana*), tomato (*Solanum lycopersicum*), soybean (*Glycine max*), lettuce (*Lactuca sativa*), wild cabbage (*Brassica oleracea*), rubber tree (*Hevea brasiliensis*), wheat (*Triticum aestivum*), chickpea (*Cicer arietinum*), and rice (*Oryza sativa*).

References

1. Wang, X.; Goregaoker, S.P.; Culver, J.N. Interaction of the Tobacco Mosaic Virus Replicase Protein with a NAC Domain Transcription Factor Is Associated with the Suppression of Systemic Host Defenses. *J Virol* **2009**, *83*, 9720–9730, doi:10.1128/JVI.00941-09.
2. McLellan, H.; Boevink, P.C.; Armstrong, M.R.; Pritchard, L.; Gomez, S.; Morales, J.; Whisson, S.C.; Beynon, J.L.; Birch, P.R.J. An RxLR Effector from Phytophthora Infestans Prevents Re-Localisation of Two Plant NAC Transcription Factors from the Endoplasmic Reticulum to the Nucleus. *PLOS Pathogens* **2013**, *9*, e1003670, doi:10.1371/journal.ppat.1003670.
3. Kim, J.-G.; Stork, W.; Mudgett, M.B. Xanthomonas Type III Effector XopD Desumoylates Tomato Transcription Factor SlERF4 to Suppress Ethylene Responses and Promote Pathogen Growth. *Cell Host Microbe* **2013**, *13*, 143–154, doi:10.1016/j.chom.2013.01.006.
4. Block, A.; Toruño, T.Y.; Elowsky, C.G.; Zhang, C.; Steinbrenner, J.; Beynon, J.; Alfano, J.R. The Pseudomonas Syringae Type III Effector HopD1 Suppresses Effector-Triggered Immunity, Localizes to the Endoplasmic Reticulum, and Targets the Arabidopsis Transcription Factor NTL9. *New Phytol* **2014**, *201*, 1358–1370, doi:10.1111/nph.12626.
5. Sarris, P.F.; Duxbury, Z.; Huh, S.U.; Ma, Y.; Segonzac, C.; Sklenar, J.; Derbyshire, P.; Cevik, V.; Rallapalli, G.; Saucet, S.B.; et al. A Plant Immune Receptor Detects Pathogen Effectors That Target WRKY Transcription Factors. *Cell* **2015**, *161*, 1089–1100, doi:10.1016/j.cell.2015.04.024.
6. Qi, M.; Link, T.I.; Müller, M.; Hirschburger, D.; Pudake, R.N.; Pedley, K.F.; Braun, E.; Voegele, R.T.; Baum, T.J.; Whitham, S.A. A Small Cysteine-Rich Protein from the Asian Soybean Rust Fungus, Phakopsora Pachyrhizi, Suppresses Plant Immunity. *PLoS Pathog* **2016**, *12*, e1005827, doi:10.1371/journal.ppat.1005827.
7. Yang, L.; Teixeira, P.J.P.L.; Biswas, S.; Finkel, O.M.; He, Y.; Salas-Gonzalez, I.; English, M.E.; Epple, P.; Mieczkowski, P.; Dangl, J.L. Pseudomonas Syringae Type III Effector HopBB1 Promotes Host Transcriptional Repressor Degradation to Regulate Phytohormone Responses and Virulence. *Cell Host Microbe* **2017**, *21*, 156–168, doi:10.1016/j.chom.2017.01.003.
8. Turnbull, D.; Yang, L.; Naqvi, S.; Breen, S.; Welsh, L.; Stephens, J.; Morris, J.; Boevink, P.C.; Hedley, P.E.; Zhan, J.; et al. RXLR Effector AVR2 Up-Regulates a Brassinosteroid-Responsive bHLH Transcription Factor to Suppress Immunity. *Plant Physiol* **2017**, *174*, 356–369, doi:10.1104/pp.16.01804.
9. Qin, J.; Wang, K.; Sun, L.; Xing, H.; Wang, S.; Li, L.; Chen, S.; Guo, H.-S.; Zhang, J. The Plant-Specific Transcription Factors CBP60g and SARD1 Are Targeted by a Verticillium Secretory Protein VdSCP41 to Modulate Immunity. *Elife* **2018**, *7*, e34902, doi:10.7554/eLife.34902.
10. Meisrimler, C.-N.; Pelgrom, A.J.E.; Oud, B.; Out, S.; Van den Ackerveken, G. Multiple Downy Mildew Effectors Target the Stress-Related NAC Transcription Factor LsNAC069 in Lettuce. *Plant J* **2019**, *99*, 1098–1115, doi:10.1111/tpj.14383.
11. Chen, S.; Ma, T.; Song, S.; Li, X.; Fu, P.; Wu, W.; Liu, J.; Gao, Y.; Ye, W.; Dry, I.B.; et al. Arabidopsis Downy Mildew Effector HaRxLL470 Suppresses Plant Immunity by Attenuating the DNA-Binding Activity of bZIP Transcription Factor HY5. *New Phytol* **2021**, *230*, 1562–1577, doi:10.1111/nph.17280.
12. Qi, P.; Huang, M.; Hu, X.; Zhang, Y.; Wang, Y.; Li, P.; Chen, S.; Zhang, D.; Cao, S.; Zhu, W.; et al. A Ralstonia Solanacearum Effector Targets TGA Transcription Factors to Subvert Salicylic Acid Signaling. *Plant Cell* **2022**, *34*, 1666–1683, doi:10.1093/plcell/koac015.
13. Zlobin, N.; Lebedeva, M.; Monakhova, Y.; Ustinova, V.; Taranov, V. An ERF121 Transcription Factor from Brassica Oleracea Is a Target for the Conserved TAL-Effectors from Different Xanthomonas Campestris Pv. Campestris Strains. *Mol Plant Pathol* **2021**, *22*, 618–624, doi:10.1111/mpp.13048.
14. Huang, W.; MacLean, A.M.; Sugio, A.; Maqbool, A.; Busscher, M.; Cho, S.-T.; Kamoun, S.; Kuo, C.-H.; Immink, R.G.H.; Hogenhout, S.A. Parasitic Modulation of Host Development by Ubiquitin-Independent Protein Degradation. *Cell* **2021**, *184*, 5201-5214.e12, doi:10.1016/j.cell.2021.08.029.
15. Strohmayer, A.; Schwarz, T.; Braun, M.; Krczal, G.; Boonrod, K. The Effect of the Anticipated Nuclear Localization Sequence of "Candidatus Phytoplasma Mali" SAP11-like Protein on Localization of the Protein and Destabilization of TCP Transcription Factor. *Microorganisms* **2021**, *9*, 1756, doi:10.3390/microorganisms9081756.

16. Yang, G.; Yang, J.; Zhang, Q.; Wang, W.; Feng, L.; Zhao, L.; An, B.; Wang, Q.; He, C.; Luo, H. The Effector Protein CgNLP1 of Colletotrichum Gloeosporioides Affects Invasion and Disrupts Nuclear Localization of Necrosis-Induced Transcription Factor HbMYB8-Like to Suppress Plant Defense Signaling. *Front. Microbiol.* **2022**, *13*, doi:10.3389/fmicb.2022.911479.
17. Zheng, P.; Liu, M.; Pang, L.; Sun, R.; Yao, M.; Wang, X.; Kang, Z.; Liu, J. Stripe Rust Effector Pst21674 Compromises Wheat Resistance by Targeting Transcription Factor TaASR3. *Plant Physiol* **2023**, *193*, 2806–2824, doi:10.1093/plphys/kiad497.
18. Singh, S.K.; Shree, A.; Verma, S.; Singh, K.; Kumar, K.; Srivastava, V.; Singh, R.; Saxena, S.; Singh, A.P.; Pandey, A.; et al. The Nuclear Effector ArPEC25 from the Necrotrophic Fungus Ascochyta Rabiei Targets the Chickpea Transcription Factor CaβLIM1a and Negatively Modulates Lignin Biosynthesis, Increasing Host Susceptibility. *Plant Cell* **2023**, *35*, 1134–1159, doi:10.1093/plcell/koac372.
19. Drcelic, M.; Skiljaica, A.; Polak, B.; Bauer, N.; Seruga Music, M. "Candidatus Phytoplasma Solani" Predicted Effector SAP11-like Alters Morphology of Transformed Arabidopsis Plants and Interacts with AtTCP2 and AtTCP4 Plant Transcription Factors. *Pathogens* **2024**, *13*, 893, doi:10.3390/pathogens13100893.
20. Yang, T.; Song, L.; Hu, J.; Qiao, L.; Yu, Q.; Wang, Z.; Chen, X.; Lu, G.-D. Magnaporthe Oryzae Effector AvrPik-D Targets a Transcription Factor WG7 to Suppress Rice Immunity. *Rice (N Y)* **2024**, *17*, 14, doi:10.1186/s12284-024-00693-0.
21. Liu, L.; Li, J.; Wang, Z.; Zhou, H.; Wang, Y.; Qin, W.; Duan, H.; Zhao, H.; Ge, X. Suppression of Plant Immunity by Verticillium Dahliae Effector Vd6317 through AtNAC53 Association. *Plant J* **2024**, *119*, 1767–1781, doi:10.1111/tpj.16883.
